# Supplementary material for: The DDHD2-STXBP1 interaction mediates long-term memory via generation of saturated free fatty acids
Source: EMBO J. 2024 Feb 5;43(4):533–67. doi: 10.1038/s44318-024-00030-7 (PMC10897203; doi:10.1038/s44318-024-00030-7)
Supplement: Supplementary file 1 — Appendix [file 44318_2024_30_MOESM1_ESM.pdf]

Appendix

The DDHD2-STXBP1 interaction mediates long-term memory via generation of saturated free fatty acids

Isaac O. Akefe[1,2*], Saber H. Saber[1,3*], Benjamin Matthews[1*], Bharat G. Venkatesh[1], Rachel S. Gormal[1], Daniel G. Blackmore[1], Suzy Alexander[1], Emma Sierecki[4,5], Yann Gambin[4,5], Jesus Bertran-Gonzalez[6], Nicolas Vitale[7], Yann Humeau[8], Arnaud Gaudin[1], Sevannah A. Ellis[1], Alysee A. Michaels[9,10], Mingshan Xue[9,10,11], Benjamin Cravatt[12], Merja Joensuu[1,3**], Tristan P. Wallis[1**] & Frédéric A. Meunier[1,13**]

[1]Clem Jones Centre for Ageing Dementia Research, Queensland Brain Institute, The University of Queensland, St Lucia, QLD, 4072, Australia

[2]Academy for Medical Education, Medical School, The University of Queensland, 288 Herston Road, 4006, Brisbane, QLD, Australia

[3]Australian Institute for Bioengineering and Nanotechnology, The University of Queensland, Brisbane, St Lucia, QLD, 4072, Australia

[4]School of Medical Science, University of New South Wales, Randwick, NSW, 2052, Australia

[5]EMBL Australia, Single Molecule Node, University of New South Wales, Sydney, 2052, Australia

[6]Decision Neuroscience Lab, School of Psychology, UNSW Sydney, Australia

[7]Institut des Neurosciences Cellulaires et Intégratives, UPR-3212 CNRS - Université de Strasbourg France

[8]Interdisciplinary Institute for Neuroscience, CNRS UMR 5297, Université de Bordeaux, France

[9]Department of Neuroscience, Baylor College of Medicine, Houston, TX, United States

[10]The Cain Foundation Laboratories, Jan and Dan Duncan Neurological Research Institute at Texas Children's Hospital, Houston, TX, United States

[11]Department of Molecular and Human Genetics, Baylor College of Medicine, Houston, TX, United States

[12]The Department of Chemistry and The Skaggs Institute for Chemical Biology, The Scripps Research Institute, La Jolla, CA, United States

[13]The School of Biomedical Sciences, The University of Queensland, St Lucia, QLD, 4072, Australia

*Equal contribution
**Corresponding authors

Correspondence to:

Frédéric A. Meunier, Tristan P. Wallis and Merja Joensuu

Email: f.meunier@uq.edu.au, t.wallis@uq.edu.au, m.joensuu@uq.edu.au

Table of Contents

Appendix Figure S1. Average FFA response to instrumental conditioning in *DDHD2*$^{+/+}$ vs *DDHD2*$^{-/-}$ mice. 3

Appendix Figure S2. FFAs and phospholipids response to instrumental conditioning in *DDHD2*$^{+/+}$ vs *DDHD2*$^{-/-}$ mice at 12mo. 4

Appendix Figure S3. Deletion of PLD1 gene is associated with heightened fear response 5

Appendix Figure S4. Phospholipid and FFA response to PLD1 knockout in contextual fear conditioning in mice. 6

Appendix Figure S5. STXBP1 interacts with DDHD2. 7

Appendix Figure S6. STXBP1 controls the activity-dependent response to exocytosis. 9

Appendix Figure S7. Profile of FFAs in PC12 cells, STXBP1 single knockout (MKO) PC12 cells, and STXBP1/2 double knockout (DKO) PC12 cells following stimulation. 10

Appendix Figure S8. Assessing the protein level of STXBP1 and DDHD2 from STXBP1 haploinsufficient mouse brains. 11

Appendix Figure S9. FFA profile of STXBP1 heterozygote mutant mice. 12

Appendix Table S1, Related to Figure 1. Multiple reaction monitoring (MRM) transitions for the liquid chromatography tandem mass spectrometry (LC-MS/MS) of FFAST derivatized free fatty acids. 13

Appendix Table S2 Related to Figure 1. Chromatographic retention times (mins) of FFAST derivatized free fatty acids. 14

Appendix Table S3 Related to Figure 1. Multiple Reaction Monitoring (MRM) transition and chromatographic retention times (RT) of FFAST reagents. 15

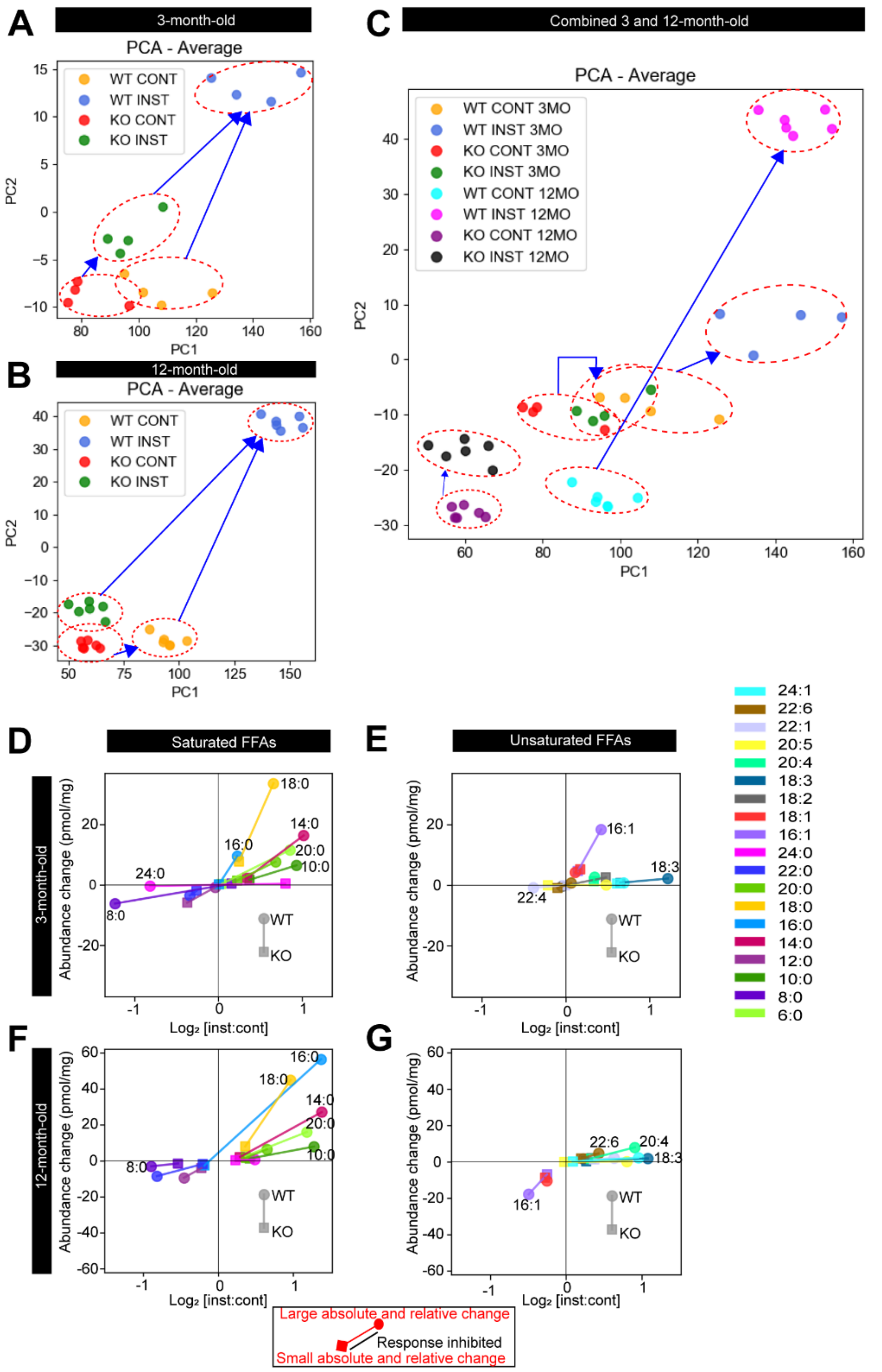

Appendix Figure S1. Average FFA response to instrumental conditioning in *DDHD2*$^{+/+}$ vs *DDHD2*$^{-/-}$ mice.

Following conditioning animals were euthanised and brains were collected for FFAST analysis. The FFA profiles from all experimental animals were dimensionally reduced using PCA and clustered according to their similarity with a red dashed outline showing a clear distinction between the different groups (control WT mice, instrumental WT mice, control KO mice and instrumental KO mice) in order to show the average FFA response for each mouse. Each dot represents the normalized mean concentrations of the analytes observed across 6 animals for each condition. PCA analysis is shown for **A** 3mo mice, **B** 12mo mice, and **C** 3mo and 12mo mice combined.Linked scatterplots show the absolute change (y axis) and $\log_2$ fold change (x axis) in mean concentration for each FFA in WT (round symbol) and KO (square symbol) mice. **D** Saturated FFA and, **E** Unsaturated FFA responses to instrumental conditioning within the dorsal hippocampus of 3mo WT vs KO mouse brains (n=6). **F** Saturated FFA and, **G** Unsaturated FFA responses to instrumental conditioning in the dorsal hippocampus of 12mo WT vs KO mouse brains (n=6).

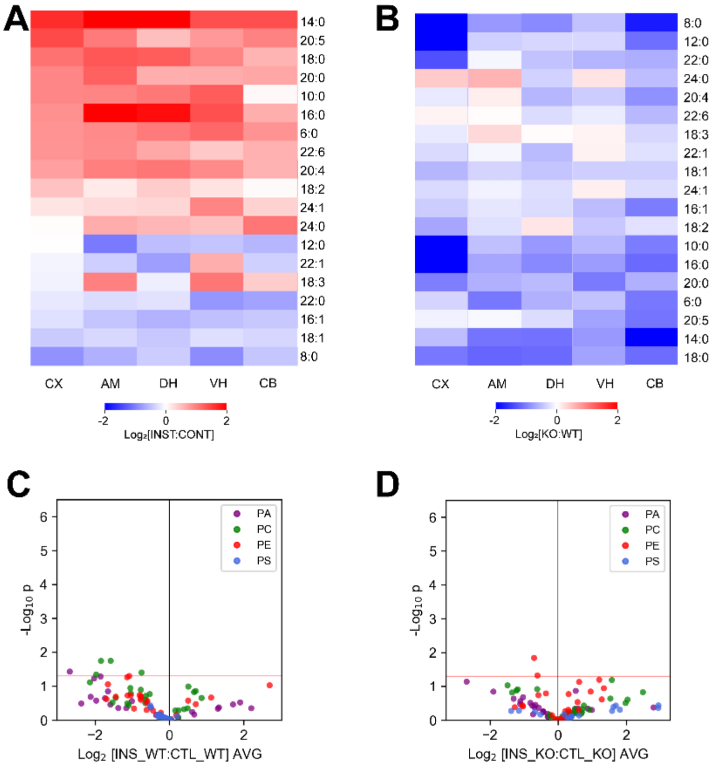

Appendix Figure S2. FFAs and phospholipids response to instrumental conditioning in *DDHD2*$^{+/+}$ vs *DDHD2*$^{-/-}$ mice at 12mo.

Heat maps showing fold change response of FFA to **A** Instrumental conditioning and **B** *DDHD2*$^{-/-}$ (KO) across different brain regions (CB; cerebellum, CX; cortex, DH; dorsal hippocampus, VH; ventral hippocampus, AM; amygdala) of wild type **(**WT) versus STXBP1$^{+/-}$ (KO) mice (n=6). Average response of phospholipids to instrumental conditioning across all the brain regions in cohorts of **C** *DDHD2*$^{+/+}$ (WT) and **D** *DDHD2*$^{-/-}$ (KO) mice: Lipids: PA - phosphatidic acid, PC - phosphatidylcholine, PE - phosphatidylethanolamine, PS – phosphatidylserine. Sample points below the red line represent those whose fold change is not significant (P > 0.05)

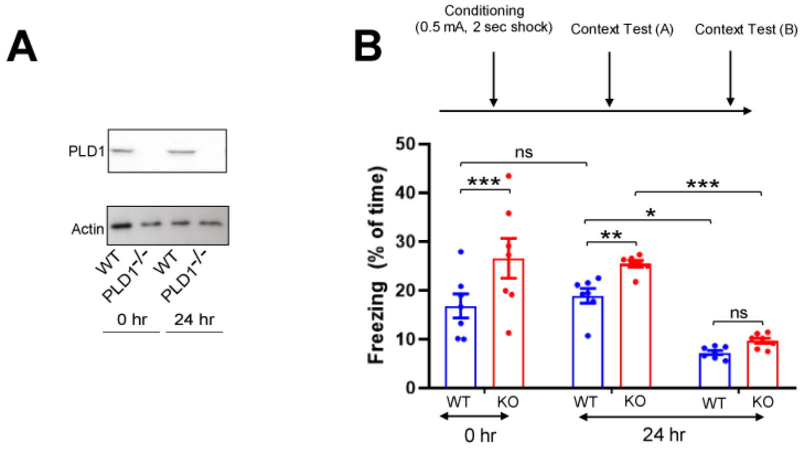

Appendix Figure S3. Deletion of PLD1 gene is associated with heightened fear response

A Protein levels of PLD1 WT and KO animals were evaluated by Western blot analysis and quantified by densitometric analysis across 5 animals (n=5). 30 µg of cellular lysate from brain of WT or PLD1 KO mice were separated on a 4-12% SDS-PAGE and probed with anti-PLD1 and actin antibodies. Deletion of PLD1 gene is associated with heightened fear response. **B** Experimental protocol generating discriminative contextual fear. WT and KO animals (n=7) were similarly conditioned using three shock applications, and then tested once for contextual fear response at different time intervals after the shock application. Freezing levels were observed during and after US pairings in WT (blue bars) or KO (red bars) mice submitted to contextual fear conditioning respectively.

Data information: In (B), two-way ANOVA: WT/KO 0h: *** $p<0.0001$; WT/KO context A 0h: ** $p=0.0014$; WT/KO context B 24hrs: ns = Not significant $p= 0.3533$, Sidak's multiple comparison test. n = 5 independent brain samples.

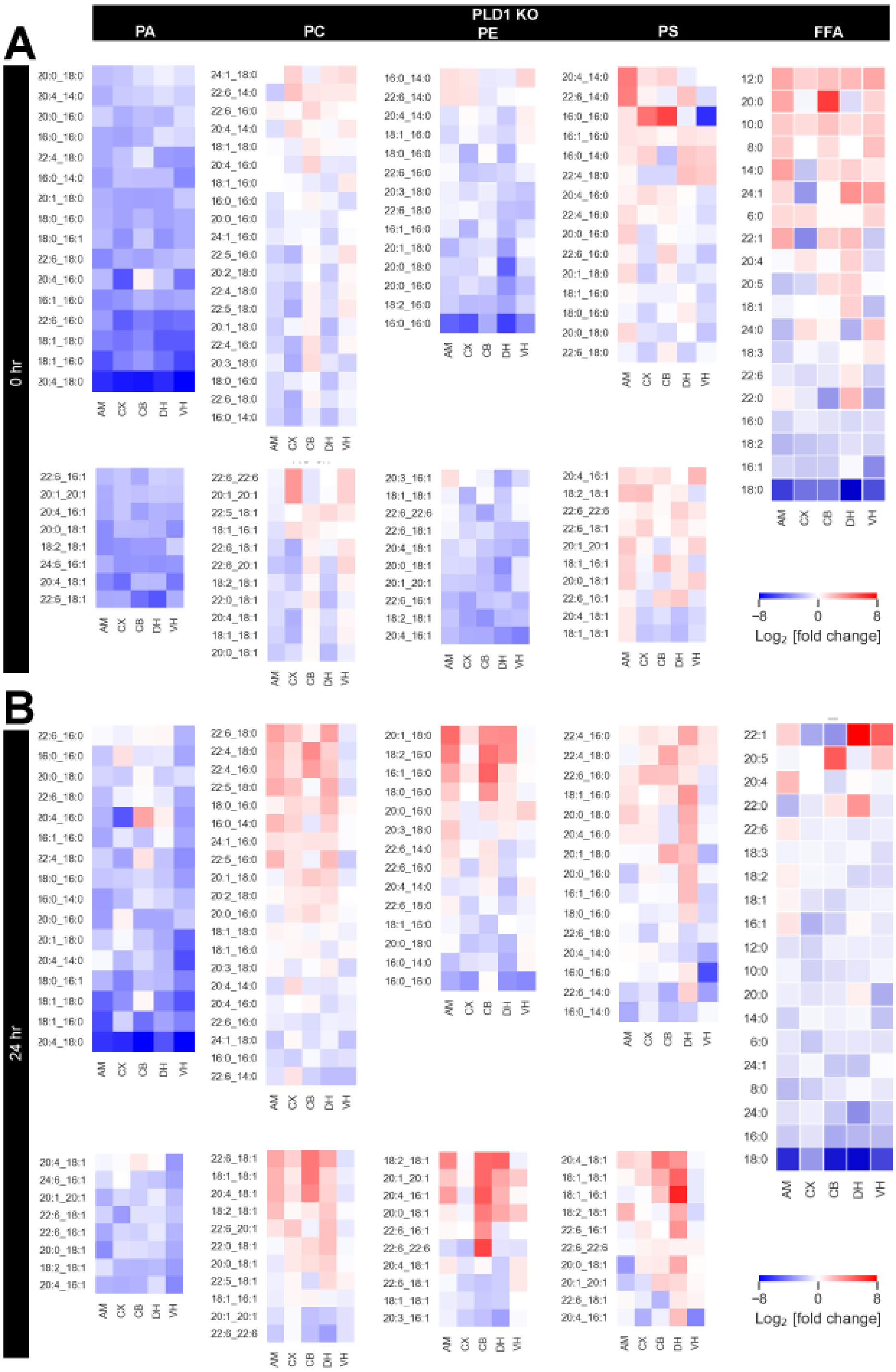

Appendix Figure S4. Phospholipid and FFA response to PLD1 knockout in contextual fear conditioning in mice.

A, B Data obtained 0hr and 24h after conditioning respectively. Data is presented as ordered heatmaps, where each pixel indicates the mean fold-change response (WT vs KO) of the corresponding analyte in each brain region.. Upper panels represent phospholipids containing C14:0, C16:0 and C18:0 acyl chains and lower panels represent phospholipids not containing C14:0, C16:0 and C18:0 acyl chains. Brain regions (CB; cerebellum, CX; cortex, DH; dorsal hippocampus, VH; ventral hippocampus, AM; amygdala).

Data information: In (A-B), white pixels represent analytes whose change in abundance was not significant (unpaired *t*-test p > 0.05). PLD1 ablation decreases the total levels of PA in all the 5 brain regions (two-tailed *t*-test $p < 0.0001$), PC (two-tailed *t*-test $p < 0.0111$), PE (two-tailed *t*-test $p < 0.0011$), PS (two-tailed *t*-test $p > 0.05$) at 0 and 24 h. n = 7 independent brain samples.

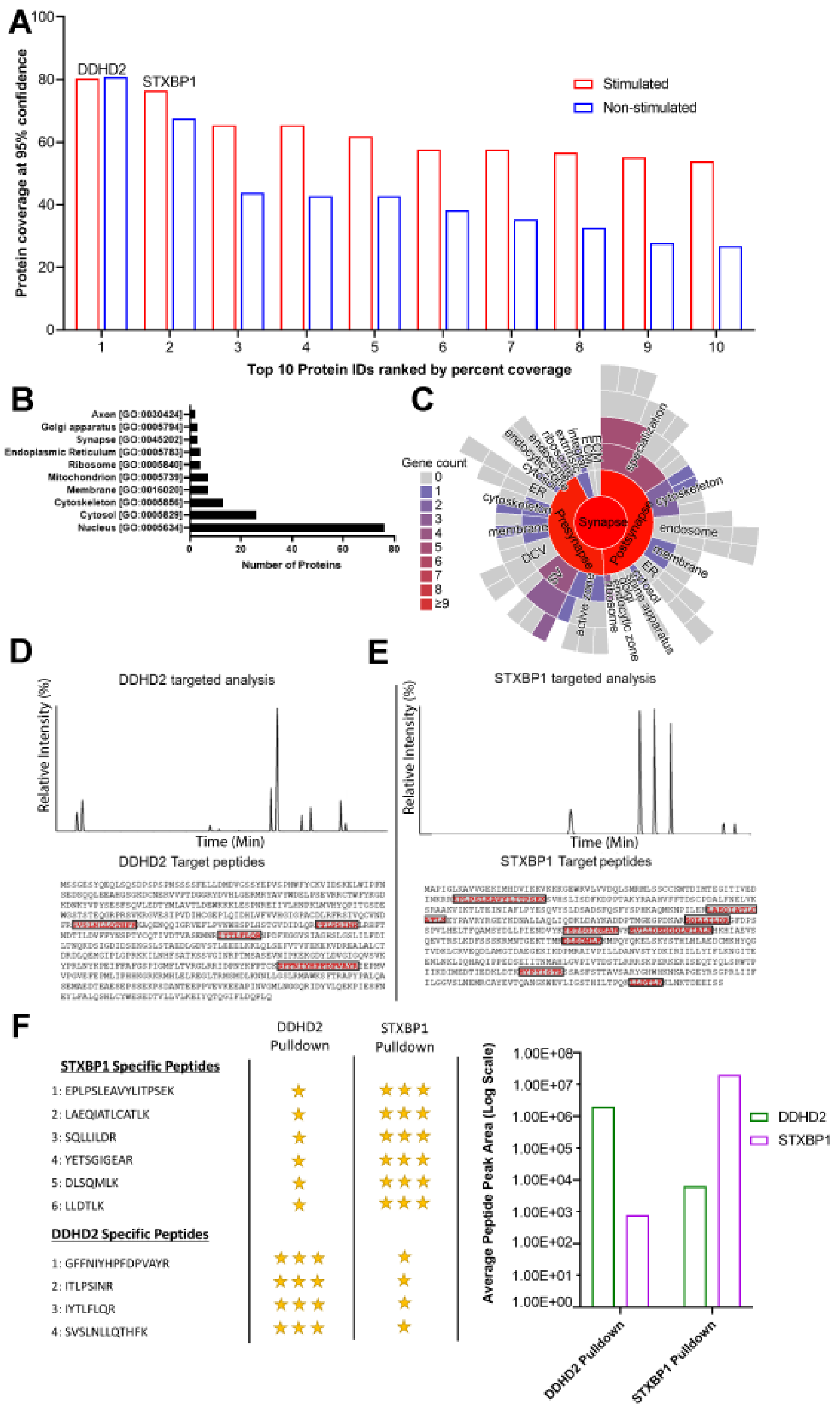

Appendix Figure S5. STXBP1 interacts with DDHD2.

Untargeted high-resolution tandem mass spectrometry (HRMS) analysis of peptide digests from a DDHD2 pull down identified 34 proteins with two or more identified peptides at 1% local false discovery rate (FDR) in resting state unstimulated PC12 cells and 214 proteins in potassium excited cells. **A** Bar plot showing the top 10 proteins co-precipitating with DDHD2 from PC12 cell lysates identified at 1% FDR ranked by identified peptides sequence coverage at 95% confidence. Using sequence coverage as an approximation of abundance Syntaxin binding protein 1 (STXBP1 the primary protein co-precipitating with DDHD2 in resting state and under potassium stimulation conditions. Cellular compartment gene ontology analysis of the identified proteins in potassium stimulated PC12 cells using the Uniprot database **B** shows that while the proteins co-precipitating

with DDHD2 are primarily associated with the nucleus and cytosol, proteins from the synapse are detecting indicating that DDHD2 may play a role in synaptic function. Further cellular compartment gene ontology analysis specific for synaptic proteins using the SynGO database **C** detected membrane and cytoskeletal proteins associated with both the pre and post synapse. Interestingly proteins, such as STXBP1, associated with synaptic vesicles (SV) and the active zone in the pre-synapse were evident confirming an interaction between DDHD2 and neuronal exocytotic mechanisms. To confirm the interaction between DDHD2 and STXBP1 an orthogonal multiple reaction monitoring (MRM) assay using triple quadrupole tandem mass spectrometry was established, specifically targeting four proteotypic DDHD2 peptides **D** and six proteotypic STXBP1 peptides **E**. The qualitative abundance for each peptide in immunoprecipitation assays targeting DDHD2 and STXBP1 in PC12 cell lysates are displayed in panel **F** confirming the presence for both proteins in both pulldowns.

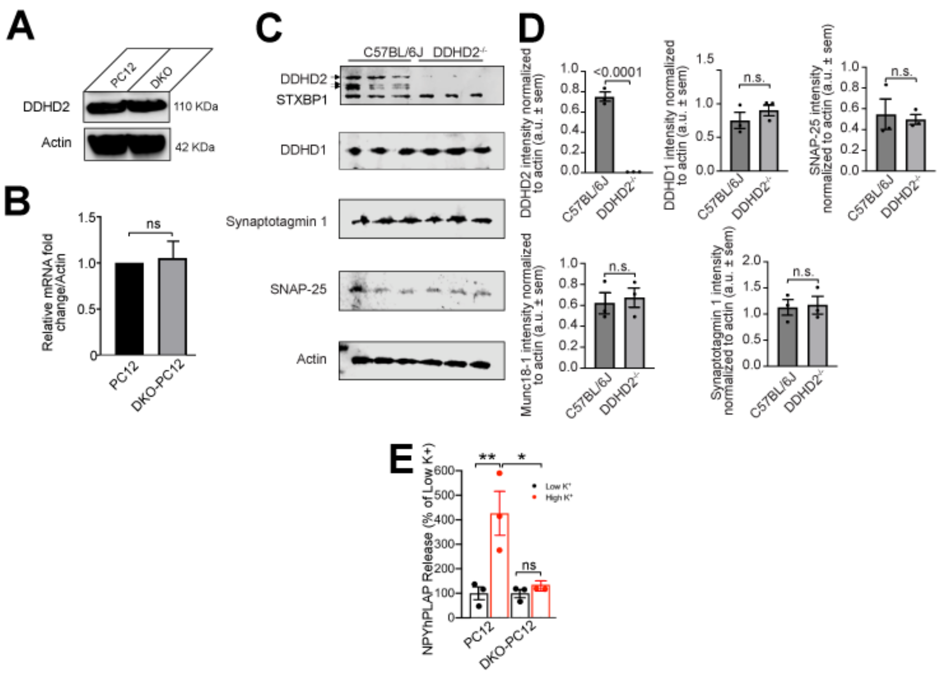

Appendix Figure S6. STXBP1 controls the activity-dependent response to exocytosis.
A Western blot and **B** RT-PCR showing levels of DDHD2 in PC12 and STXBP1/2 DKO cells. Results are shown as mean ± SEM from four independent experiments. **C** Quantification of neuropeptide-Y (NPY) release in PC12 and STXBP1/2 DKO PC12 cells co-transfected with NPY-human placental alkaline phosphatase (NPY-hPLAP) plasmid, and the indicated STXBP1 plasmid, for 72 h. DDHD2 Knock out has no impact on protein expression levels of STXBP-1, DDHD1, and synaptic markers as shown by **D** Western blot analysis and **E** quantification markers indicated in E16 C57BL/6J and DDHD2$^{-/-}$ hippocampal neurons at DIV21-22.

Data information: In (B, D, E), statistical analysis was performed using Student's *t*-test. All data are represented as mean ± SEM from 3 independent experiments. One-way ANOVA with Tukey's correction for multiple comparisons, *p<0.05, **p<0.01, ns = not significant.

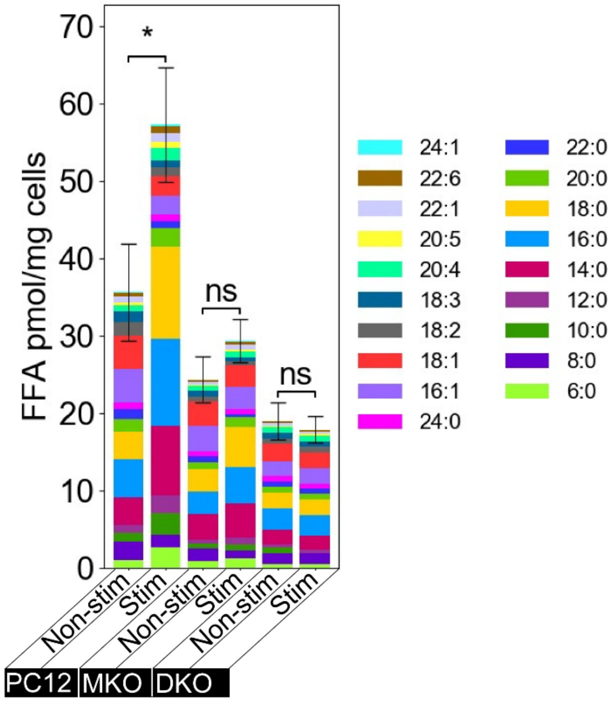

Appendix Figure S7. Profile of FFAs in PC12 cells, STXBP1 single knockout (MKO) PC12 cells, and STXBP1/2 double knockout (DKO) PC12 cells following stimulation.
PC12, MKO, and DKO cells were stimulated (Stim) by depolarization for 15 min in high K^+ (60 mM) buffer. Control unstimulated (Non-stim) cells were treated for 15 min in low K^+ (2 mM) buffer.

Data information: In (S7), the significance of the change in average FFA abundance between PC12 Non-stim/Stim, MKO Non-stim/Stim, and DKO Non-stim/Stim as determined by t-test with Holm-Sidak *post-hoc* correction is indicated by asterisks * p<0.01. ns = not significant. The error bars represent the cumulative standard error of the mean (SEM). n = 3 biological replicates.

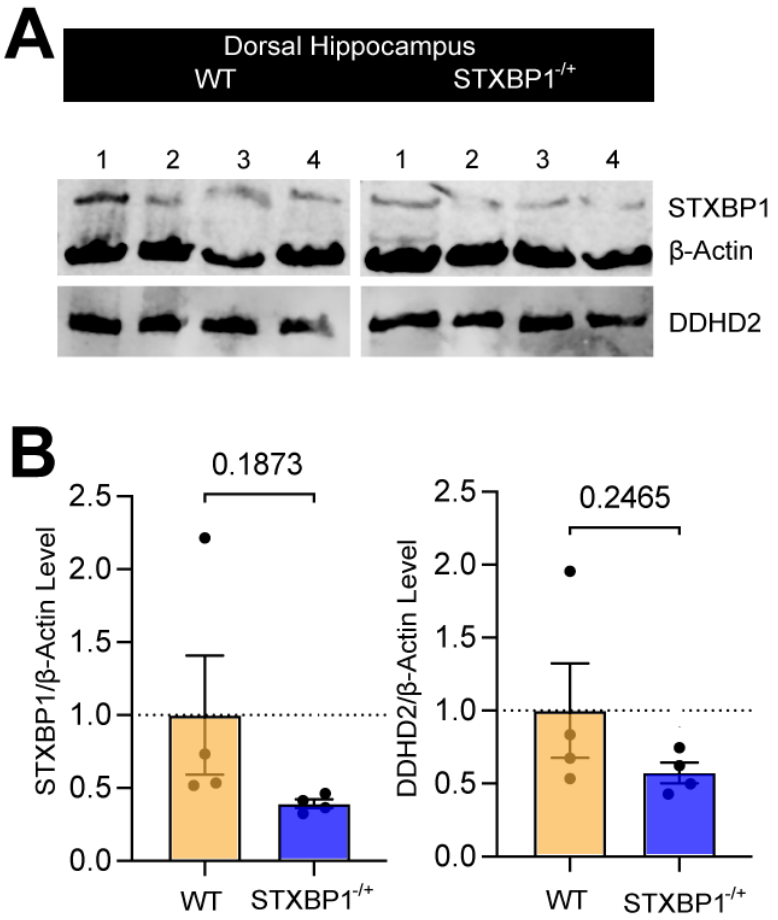

Appendix Figure S8. Assessing the protein level of STXBP1 and DDHD2 from STXBP1 haploinsufficient mouse brains.

Western blotting analysis from wild-type (WT) and STXBP1 heterozygous knockout (STXBP1$^{-/+}$) mouse brains was performed to assess the expression levels of STXBP1 and DDHD2. **A** Western blot of Dorsal Hippocampus was blotted using anti-Mouse STXBP1 and anti-β-Actin (top panels), as well as anti-Rabbit-DDHD2 antibodies (bottom panels) (1-4 represent biological replicates). **B** Analysis of the relative protein level of STXBP1 (left) or DDHD2 (right) (relative to β-Actin) from Dorsal Hippocampal samples is displayed.

Data information: In (B), Values are presented as mean ± SEM. Student's *t*-test was used to compare protein expression from WT (orange bars) vs STXBP-1$^{-/+}$ brains (blue bars). n = 4 mice for each condition.

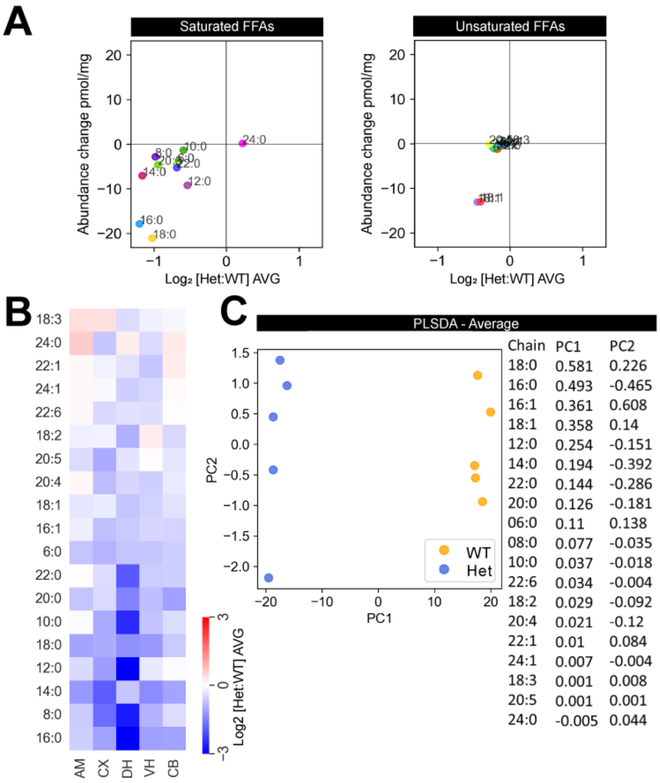

Appendix Figure S9. FFA profile of STXBP1 heterozygote mutant mice.
A Scatter plot showing absolute vs fold change of average saturated and unsaturated FFAs across the brain of WT versus heterozygote mutant mice. **B** Heatmap of the response of each FFA species in WT versus haploinsufficient STXBP1 animals. **C** Partial least square discriminant analysis (PLSDA) of the average FFA profile across the brain, with tabulated weighting of analyte contribution to clustering shown on the right.

	MRM(m/z) ions		
**Fatty acid chain**	**FFAST-124**	**FFAST-127**	**FFAST-138**
C6:0	222.1 – 124.1	225.1 – 127.1	236.1 – 138.1
C8:0	250.2 – 124.1	253.2 – 127.1	264.2 – 138.1
C10:0	278.2 – 124.1	281.2 – 127.1	292.2 – 138.1
C12:0	306.2 – 124.1	309.2 – 127.1	320.1 – 138.1
C14:0	334.3 – 124.1	337.3 – 127.1	348.1 – 138.1
C16:0	362.2 – 124.1	365.2 – 127.1	376.2 – 138.1
C18:0	390.2 – 124.1	393.2 – 127.1	404.1 – 138.1
C20:0	418.3 – 124.1	421.3 – 127.1	432.1 – 138.1
C22:0	446.2 – 124.1	449.2 – 127.1	460.2 – 138.1
C24:0	474.4 – 124.1	477.4 – 127.1	488.4 – 138.1
C16:1	360.2 – 124.1	363.2 – 127.1	374.2 – 138.1
C18:1	388.3 – 124.1	391.3 – 127.1	402.3 – 138.1
C18:2	386.2 – 124.1	389.2 – 127.1	400.2 – 138.1
C18:3	384.2 – 124.1	387.2 – 127.1	398.2 – 138.1
C20:4	410.2 – 124.1	413.2 – 127.1	424.2 – 138.1
C20:5	408.4 – 124.1	411.4 – 127.1	422.4 – 138.1
C22:1	444.3 – 124.1	444.3 – 124.1	458.3 – 138.1
C22:6	434.2 – 124.1	437.2 – 127.1	448.2 – 138.1
C24:1	472.4 – 124.1	475.4 – 127.1	486.4 – 138.1

Appendix Table S1, Related to Figure 1. Multiple reaction monitoring (MRM) transitions for the liquid chromatography tandem mass spectrometry (LC-MS/MS) of FFAST derivatized free fatty acids.

Fatty acid chain	Common name	FFAST-124	FFAST-127	FFAST-138
C6:0	Hexanoic acid	0.42	0.42	0.43
C8:0	Octanoic acid	0.48	0.46	0.47
C10:0	Decanoic acid	0.59	0.59	0.58
C12:0	Dodecanoic acid	0.89	0.88	0.87
C14:0	Tetradecanoic acid	1.56	1.55	1.55
C16:0	Hexadecanoic acid	2.85	2.83	2.84
C18:0	Octadecanoic acid	4.64	4.62	4.62
C20:0	Eicosanoic acid	5.95	5.94	5.94
C22:0	Docosanoic acid	6.75	6.74	6.74
C24:0	Lignoceric acid	7.78	7.74	7.78
C16:1	Palmitoleic acid	1.84	1.81	1.84
C18:1	Oleic acid	3.22	3.21	3.22
C18:2	Linoleic acid	2.21	2.20	2.20
C18:3	Linolenic acid	1.68	1.64	1.67
C20:4	Arachidonic acid	2.11	2.10	2.08
C20:5	Eicosapentaneoic acid	1.41	1.42	1.43
C22:1	Erucic acid	6.02	6.10	6.03
C22:6	Docosahexaenoic acid	1.99	1.98	1.99
C24:1	Nervonic acid	6.77	6.76	6.76

Appendix Table S2 Related to Figure 1. Chromatographic retention times (mins) of FFAST derivatized free fatty acids.

FFAST Reagents	MRM (m/z) ions	RT (min)
FFAST-124	124.1 – 106.2<br>124.1 – 107.1	0.24
FFAST-127	127.1 – 110.2<br>127.1 – 109.2	0.21
FFAST-138	138.1 – 106.3<br>138.1 – 121.2	0.22

Appendix Table S3 Related to Figure 1. Multiple Reaction Monitoring (MRM) transition and chromatographic retention times (RT) of FFAST reagents.
