# Supplementary figures and images for: The DDHD2-STXBP1 interaction mediates long-term memory via generation of saturated free fatty acids
Source: EMBO J. 2024 Feb 5;43(4):533–67. doi: 10.1038/s44318-024-00030-7 (PMC10897203; doi:10.1038/s44318-024-00030-7)

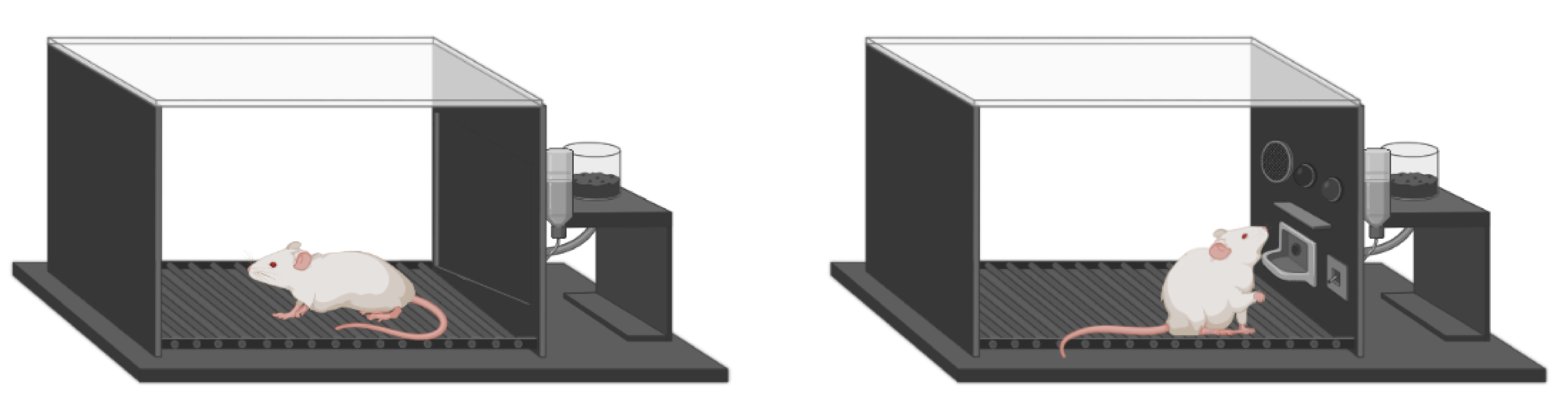

Supplement: Supplementary file 2 — Source Data Fig. 1 [file 44318_2024_30_MOESM2_ESM.zip › Figure 1/1A/Instrumental conditioning apparatus.tif]

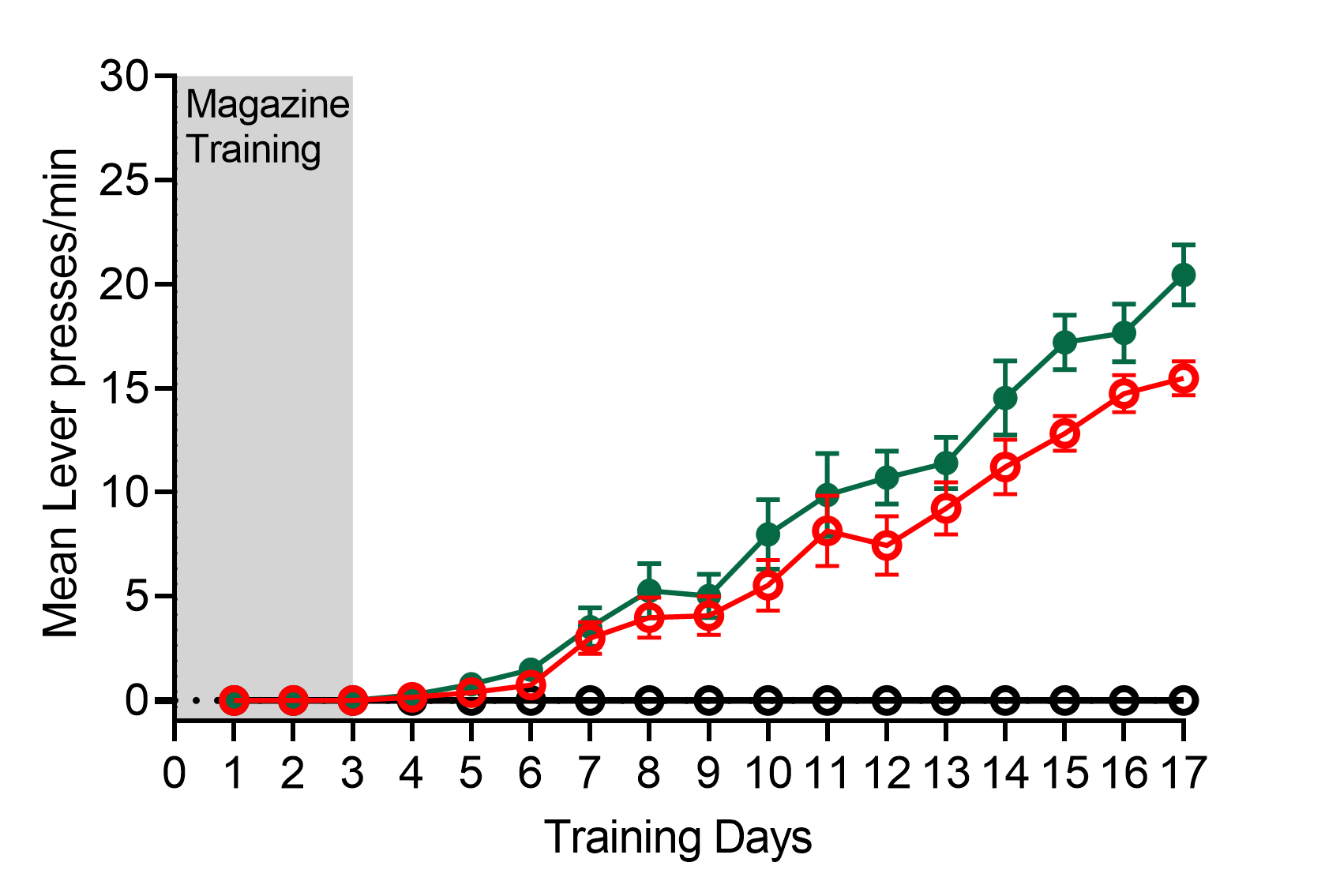

Supplement: Supplementary file 2 — Source Data Fig. 1 [file 44318_2024_30_MOESM2_ESM.zip › Figure 1/1B/Figure 1B 3MO Lever pressing.tif]

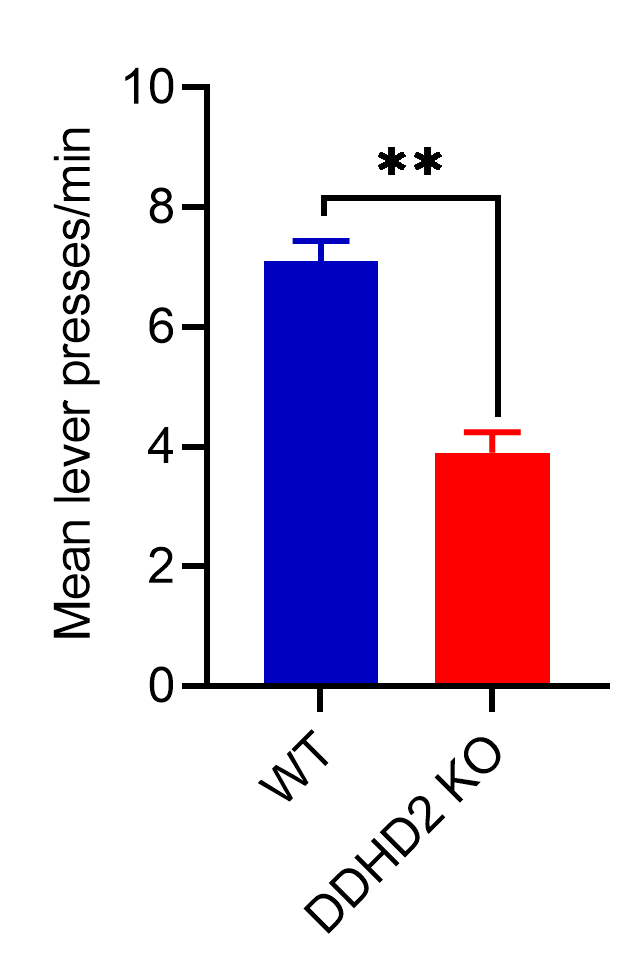

Supplement: Supplementary file 2 — Source Data Fig. 1 [file 44318_2024_30_MOESM2_ESM.zip › Figure 1/1C/Figure 1C 3MO Lever pressing.tif]

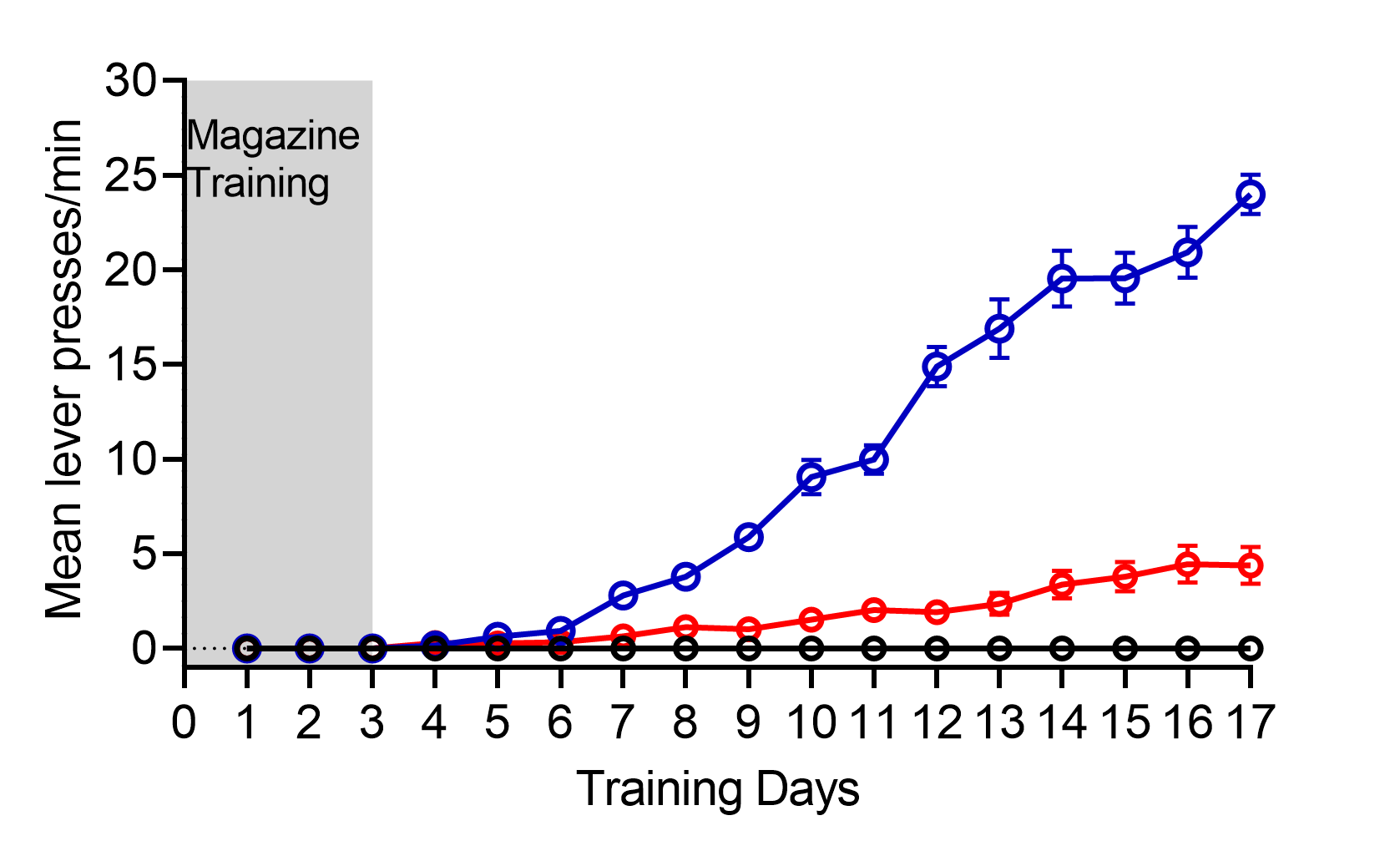

Supplement: Supplementary file 2 — Source Data Fig. 1 [file 44318_2024_30_MOESM2_ESM.zip › Figure 1/1D/Figure 1D 12 MO Lever pressing.tif]

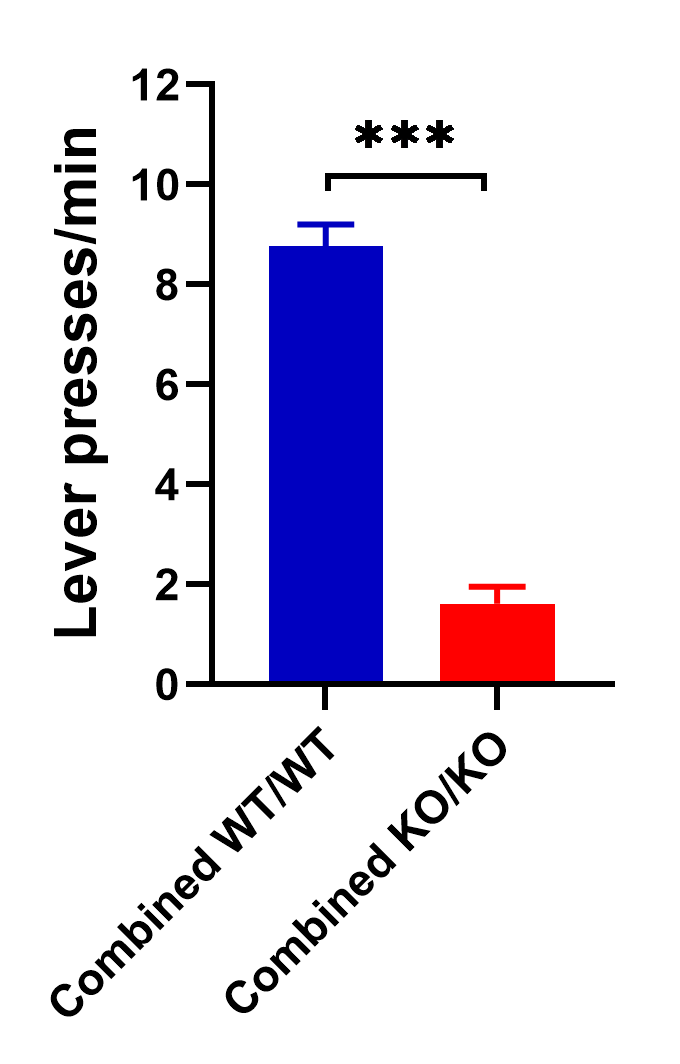

Supplement: Supplementary file 2 — Source Data Fig. 1 [file 44318_2024_30_MOESM2_ESM.zip › Figure 1/1E/Figure 1E 12 MO Lever Pressing.tif]

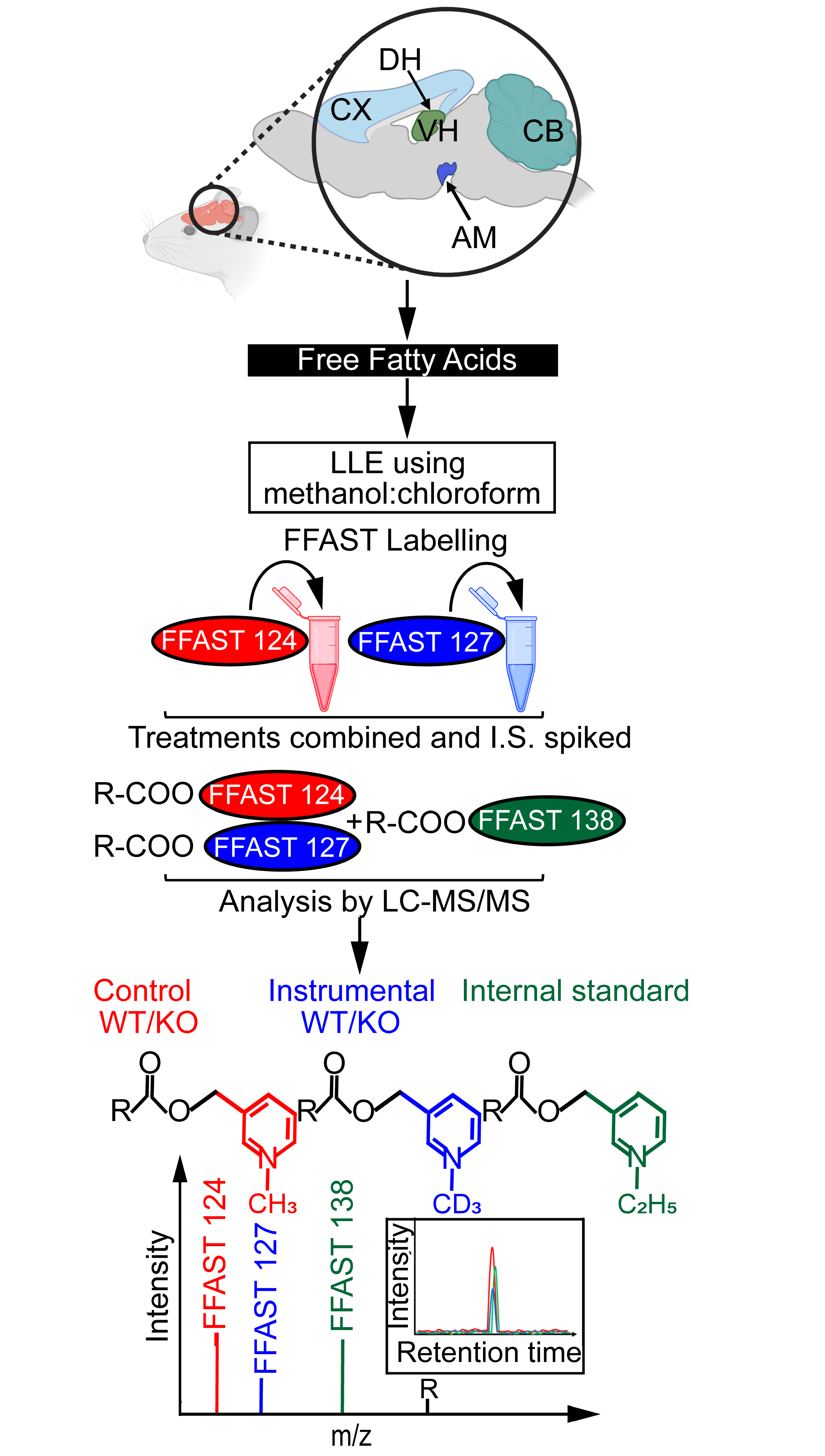

Supplement: Supplementary file 3 — Source Data Fig. 2 [file 44318_2024_30_MOESM3_ESM.zip › Figure 2/2A/Figure 2A Illustration of FFA profiling.tif]

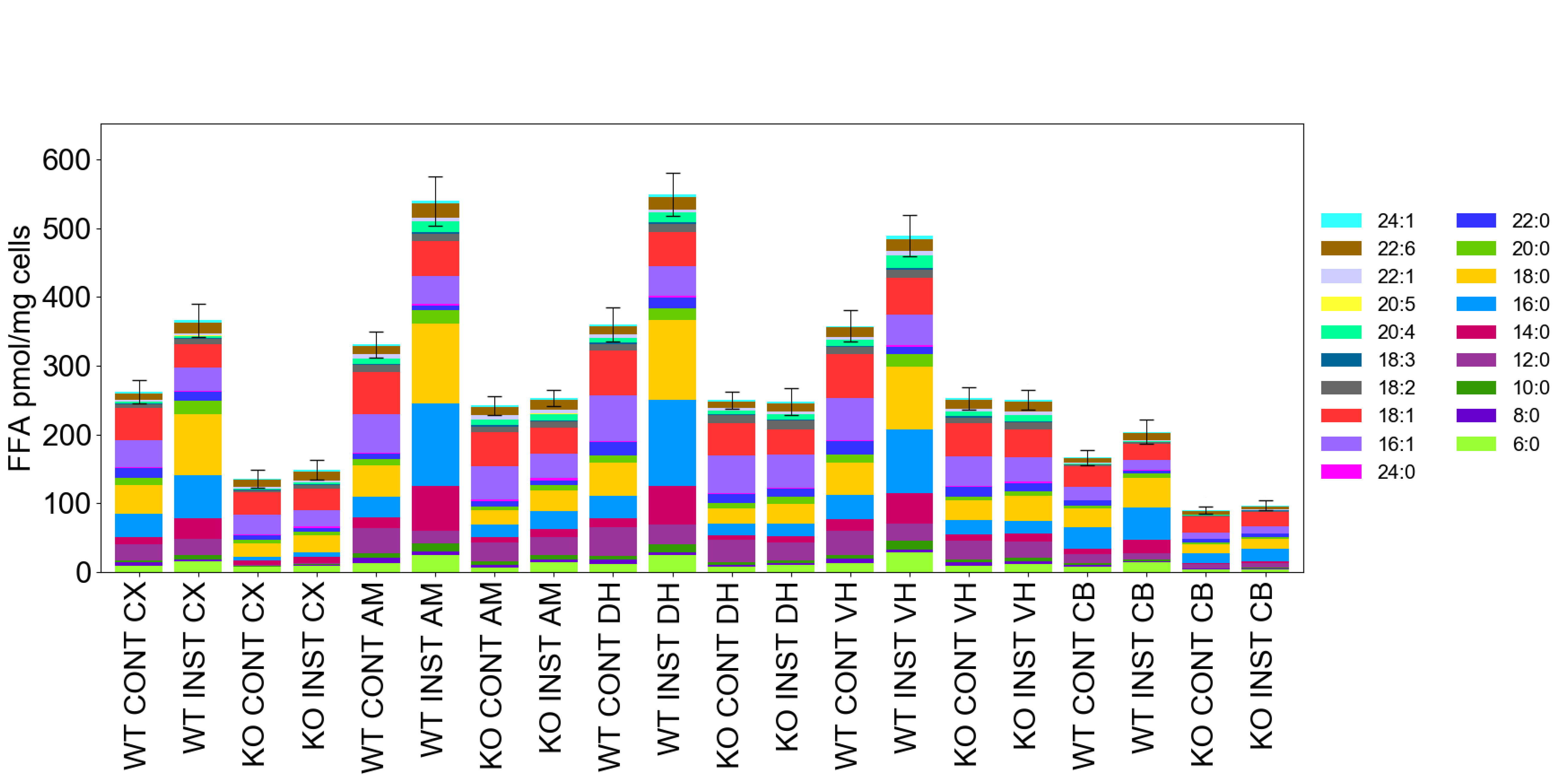

Supplement: Supplementary file 3 — Source Data Fig. 2 [file 44318_2024_30_MOESM3_ESM.zip › Figure 2/2B/Figure 2B 12MO FFA Profile.tif]

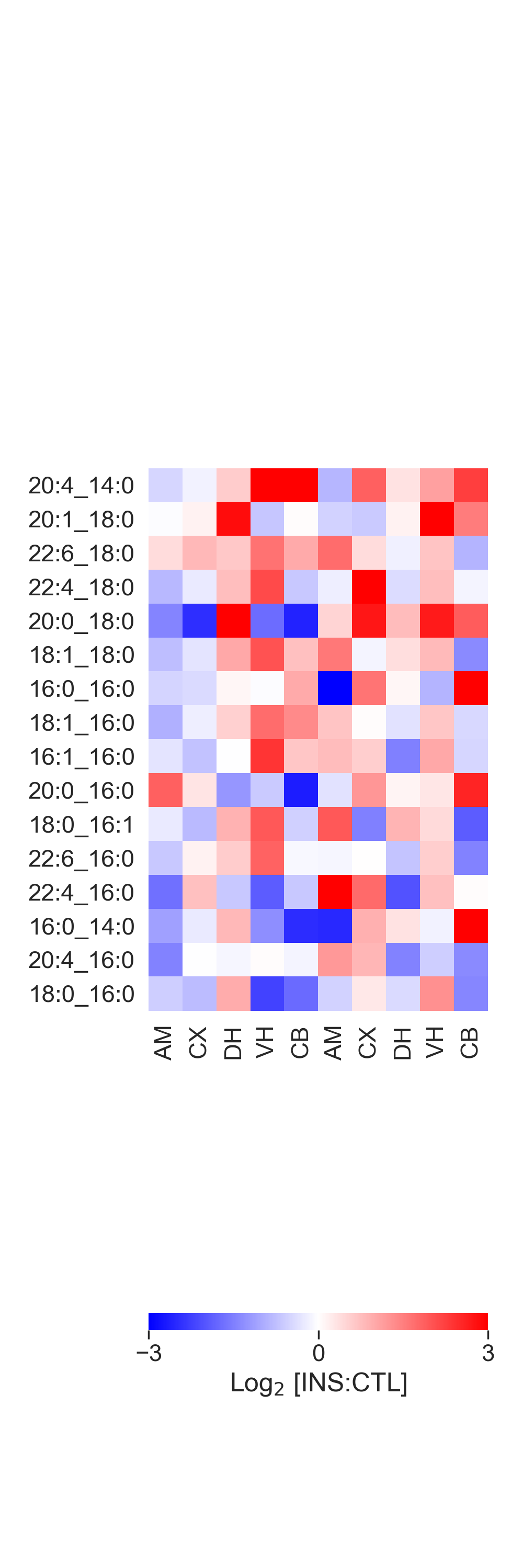

Supplement: Supplementary file 4 — Source Data Fig. 3 [file 44318_2024_30_MOESM4_ESM.zip › Figure 3/3A/heatmap_PA_20230926-130400.png]

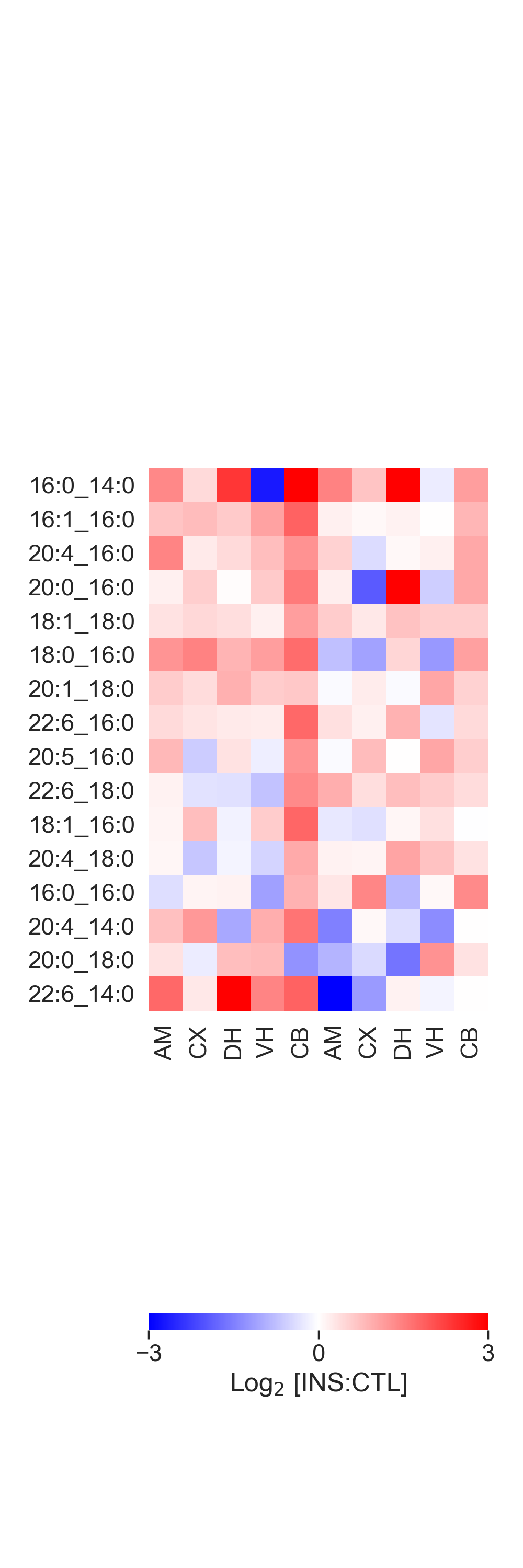

Supplement: Supplementary file 4 — Source Data Fig. 3 [file 44318_2024_30_MOESM4_ESM.zip › Figure 3/3B/heatmap_PE_20230926-130519.png]

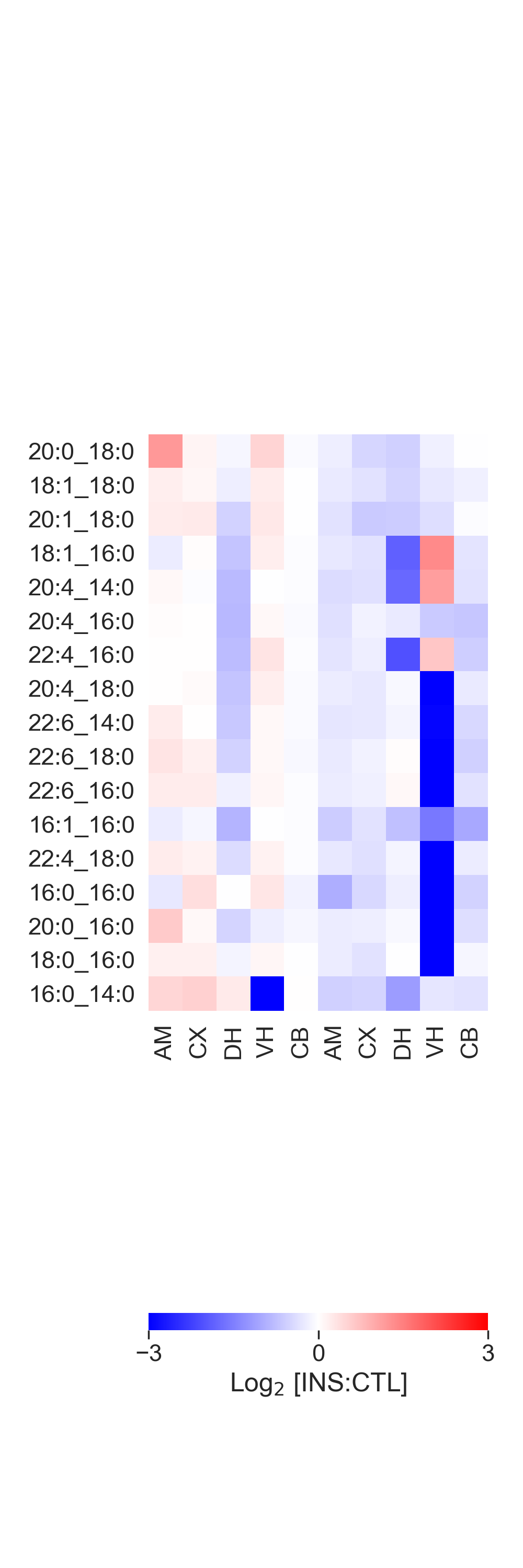

Supplement: Supplementary file 4 — Source Data Fig. 3 [file 44318_2024_30_MOESM4_ESM.zip › Figure 3/3C/heatmap_PS_20230926-130545.png]

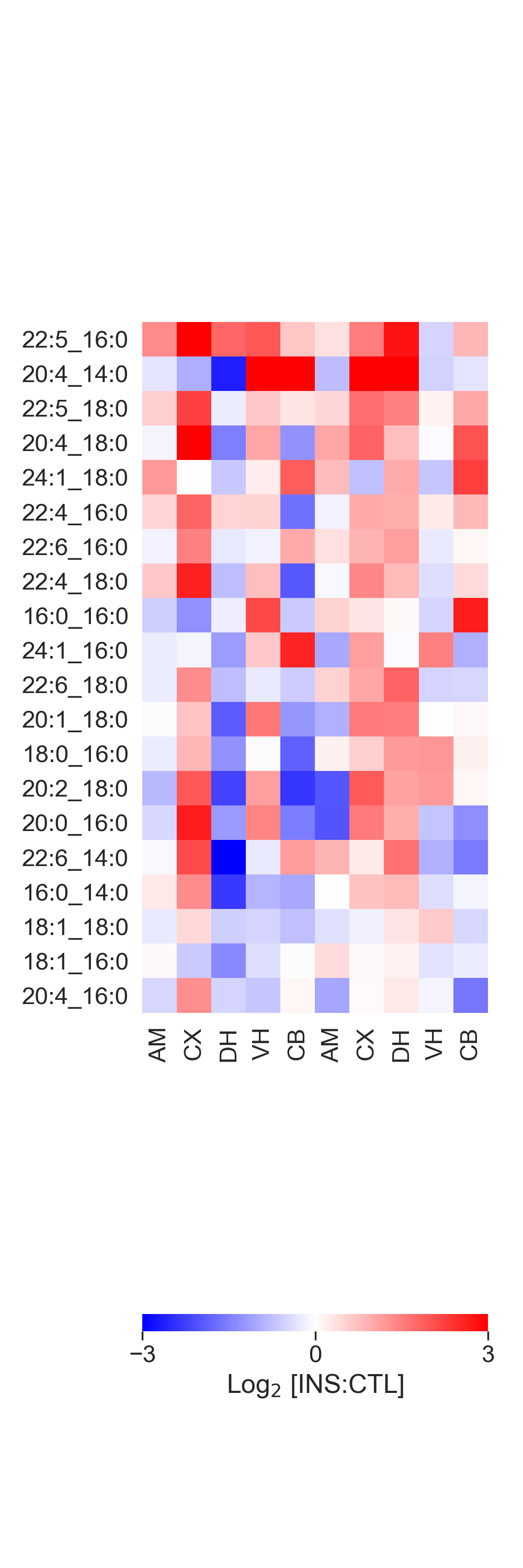

Supplement: Supplementary file 4 — Source Data Fig. 3 [file 44318_2024_30_MOESM4_ESM.zip › Figure 3/3D/heatmap_PC_20230926-130454.png]

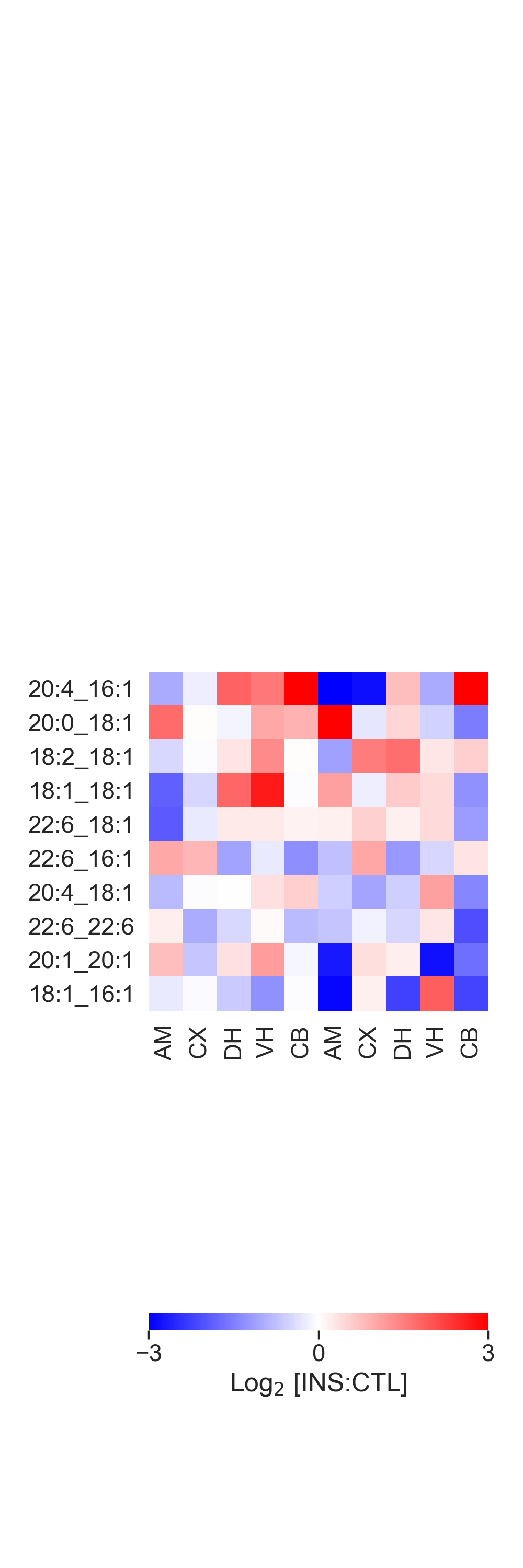

Supplement: Supplementary file 4 — Source Data Fig. 3 [file 44318_2024_30_MOESM4_ESM.zip › Figure 3/3E/heatmap_PA_20230926-131346.png]

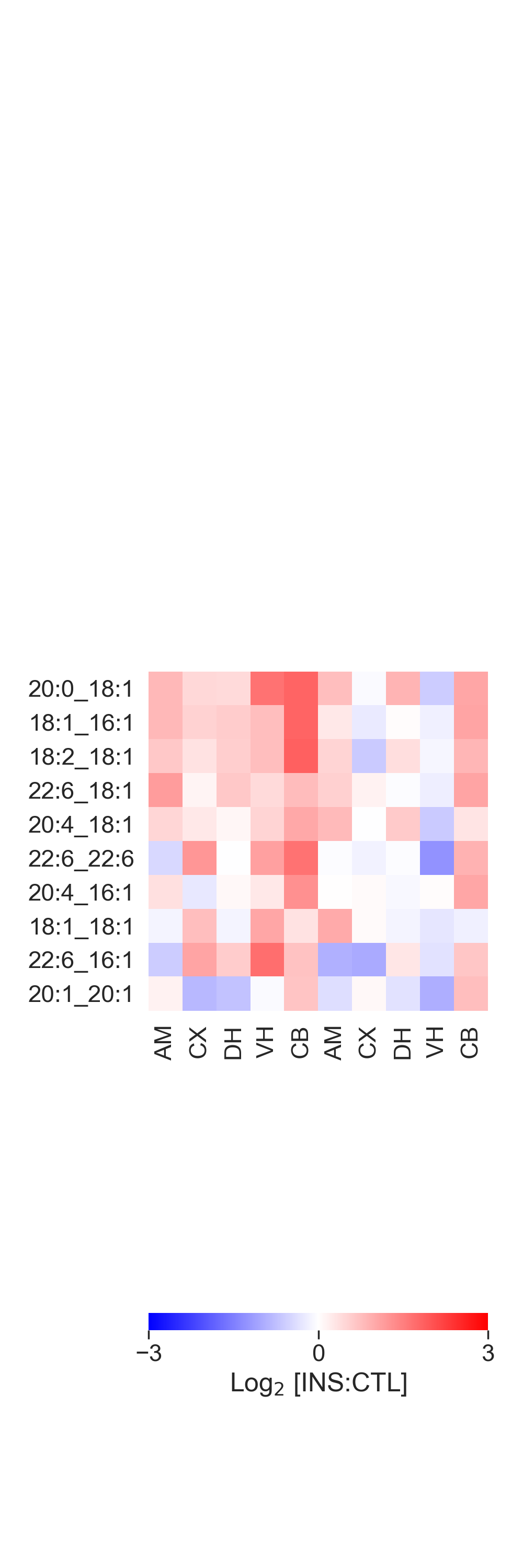

Supplement: Supplementary file 4 — Source Data Fig. 3 [file 44318_2024_30_MOESM4_ESM.zip › Figure 3/3F/heatmap_PE_20230926-131433.png]

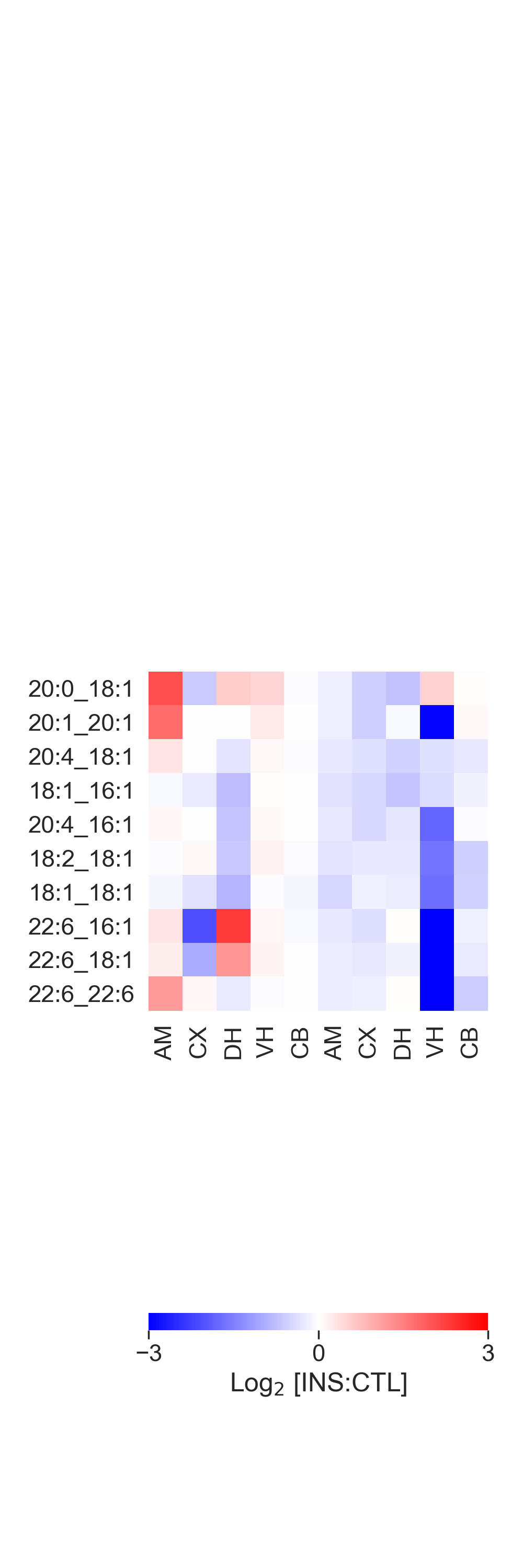

Supplement: Supplementary file 4 — Source Data Fig. 3 [file 44318_2024_30_MOESM4_ESM.zip › Figure 3/3G/heatmap_PS_20230926-131513.png]

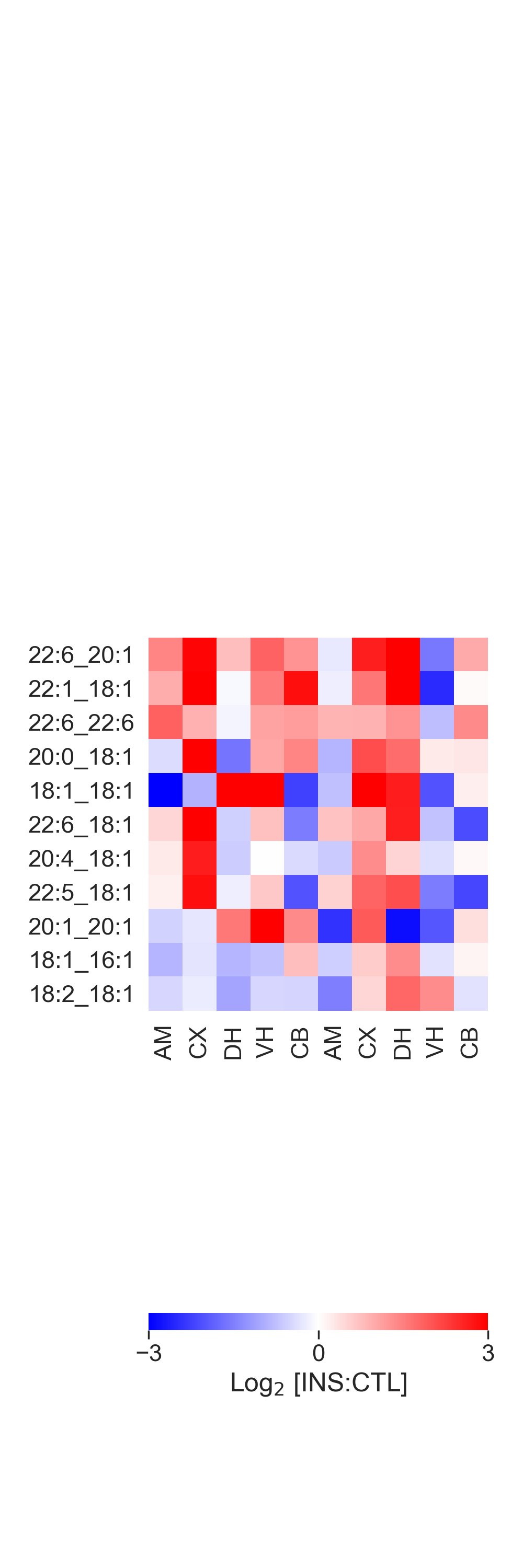

Supplement: Supplementary file 4 — Source Data Fig. 3 [file 44318_2024_30_MOESM4_ESM.zip › Figure 3/3H/heatmap_PC_20230926-131405 - Copy.png]

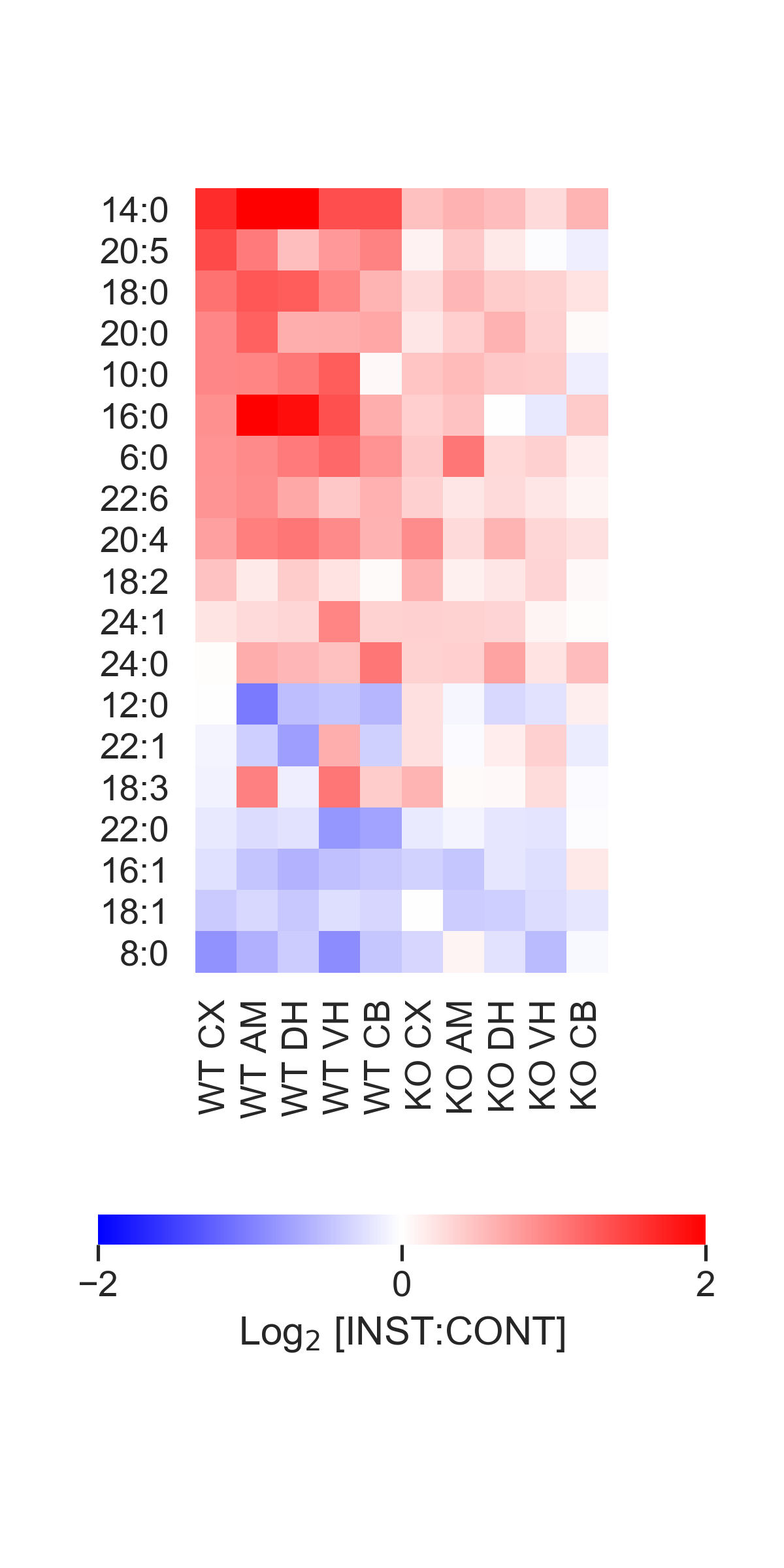

Supplement: Supplementary file 4 — Source Data Fig. 3 [file 44318_2024_30_MOESM4_ESM.zip › Figure 3/3I/20210930-143614_foldchange_heatmap.png]

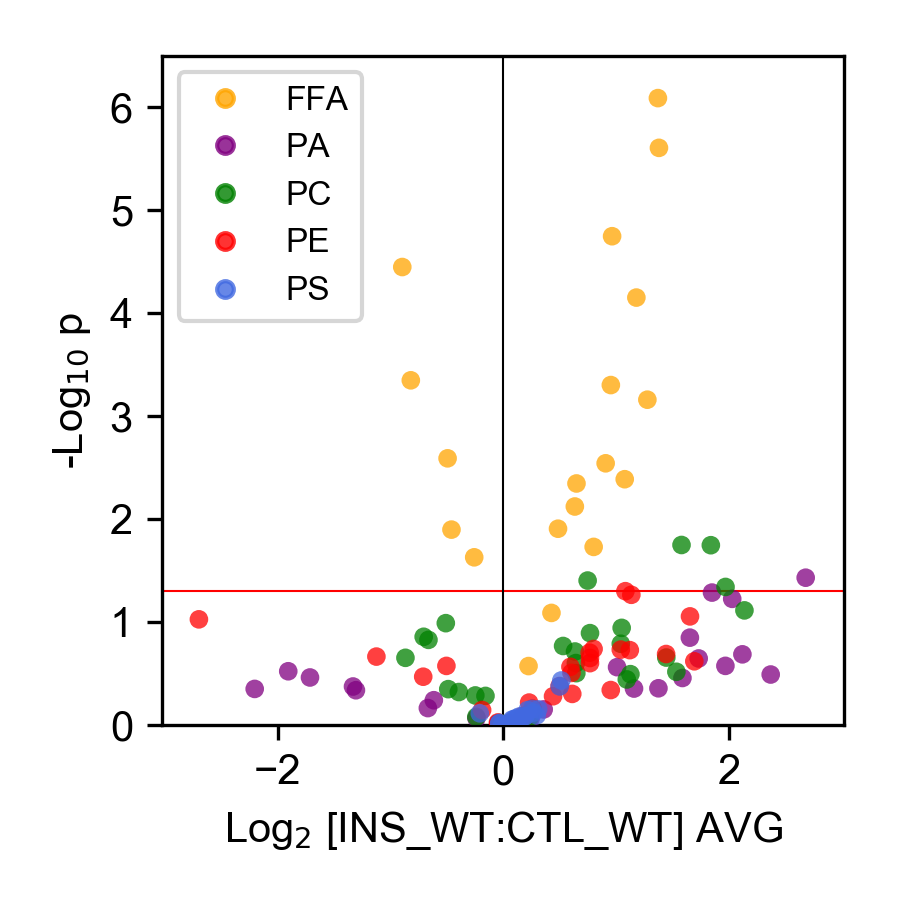

Supplement: Supplementary file 4 — Source Data Fig. 3 [file 44318_2024_30_MOESM4_ESM.zip › Figure 3/3J/volcanoplot_INS_WT_CTL_WT_AVG_20230921-112324.png]

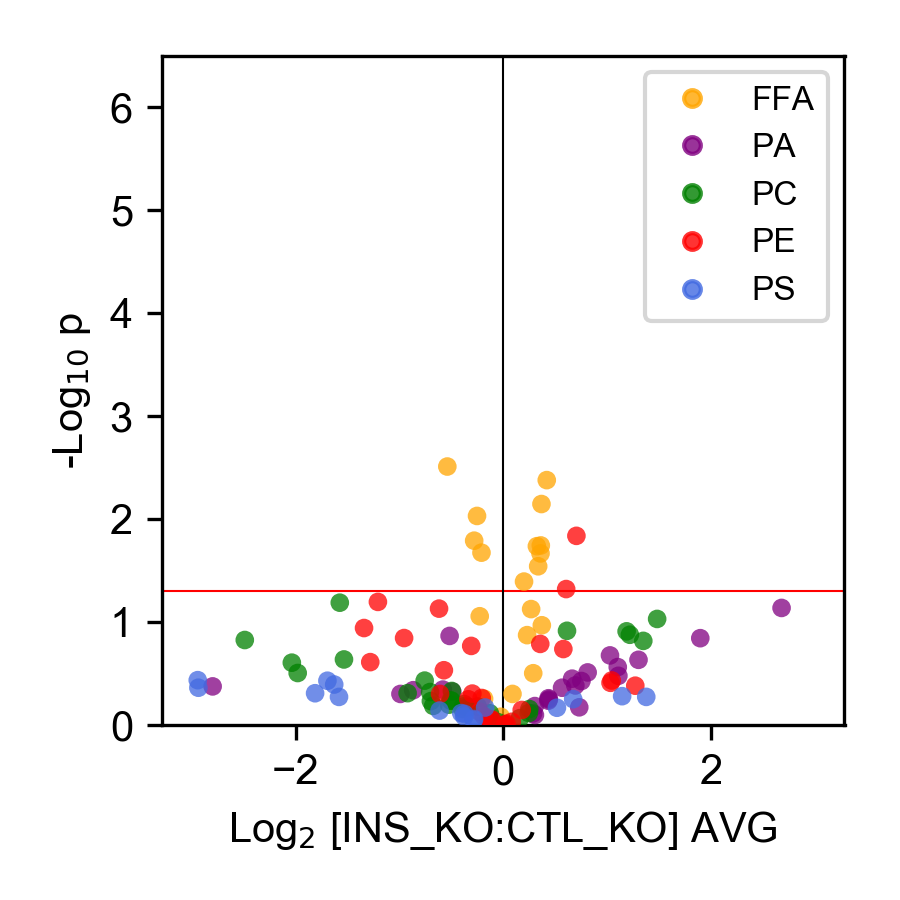

Supplement: Supplementary file 4 — Source Data Fig. 3 [file 44318_2024_30_MOESM4_ESM.zip › Figure 3/3K/volcanoplot_INS_KO_CTL_KO_AVG_20230921-112452.png]

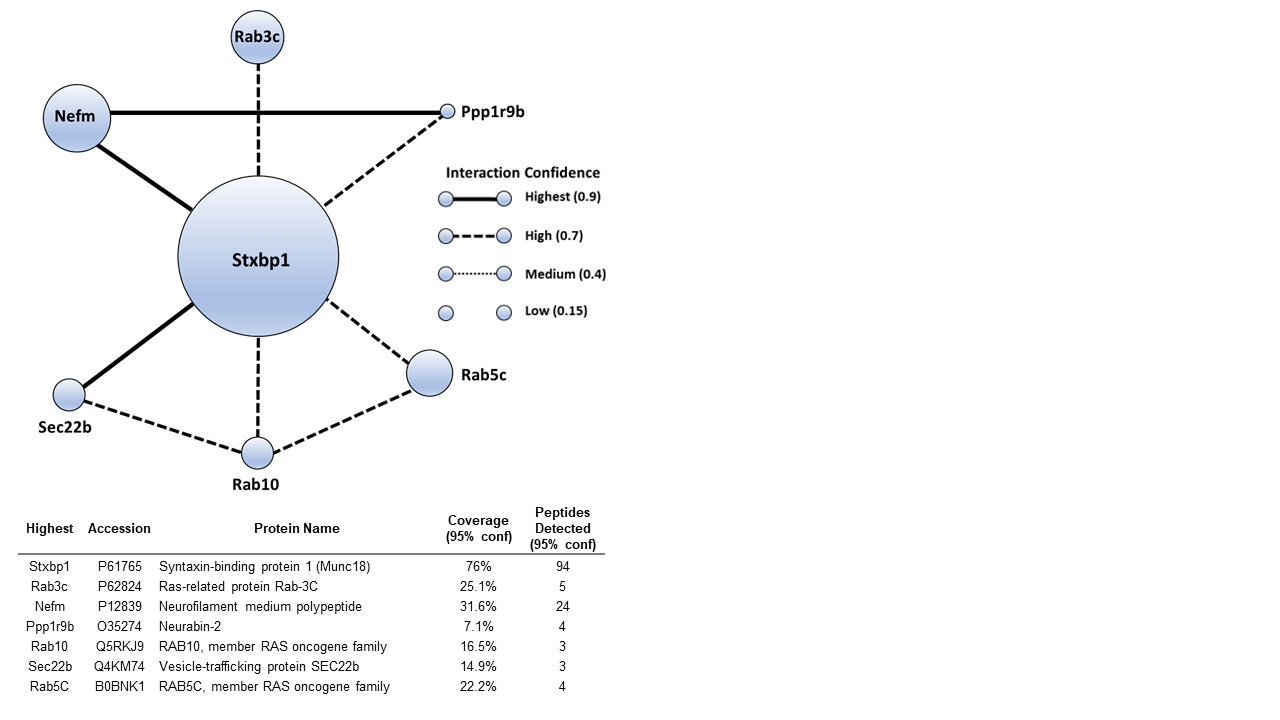

Supplement: Supplementary file 5 — Source Data Fig. 4 [file 44318_2024_30_MOESM5_ESM.zip › Figure 4/4A/Fig 4-A Munc18 interactors.tif]

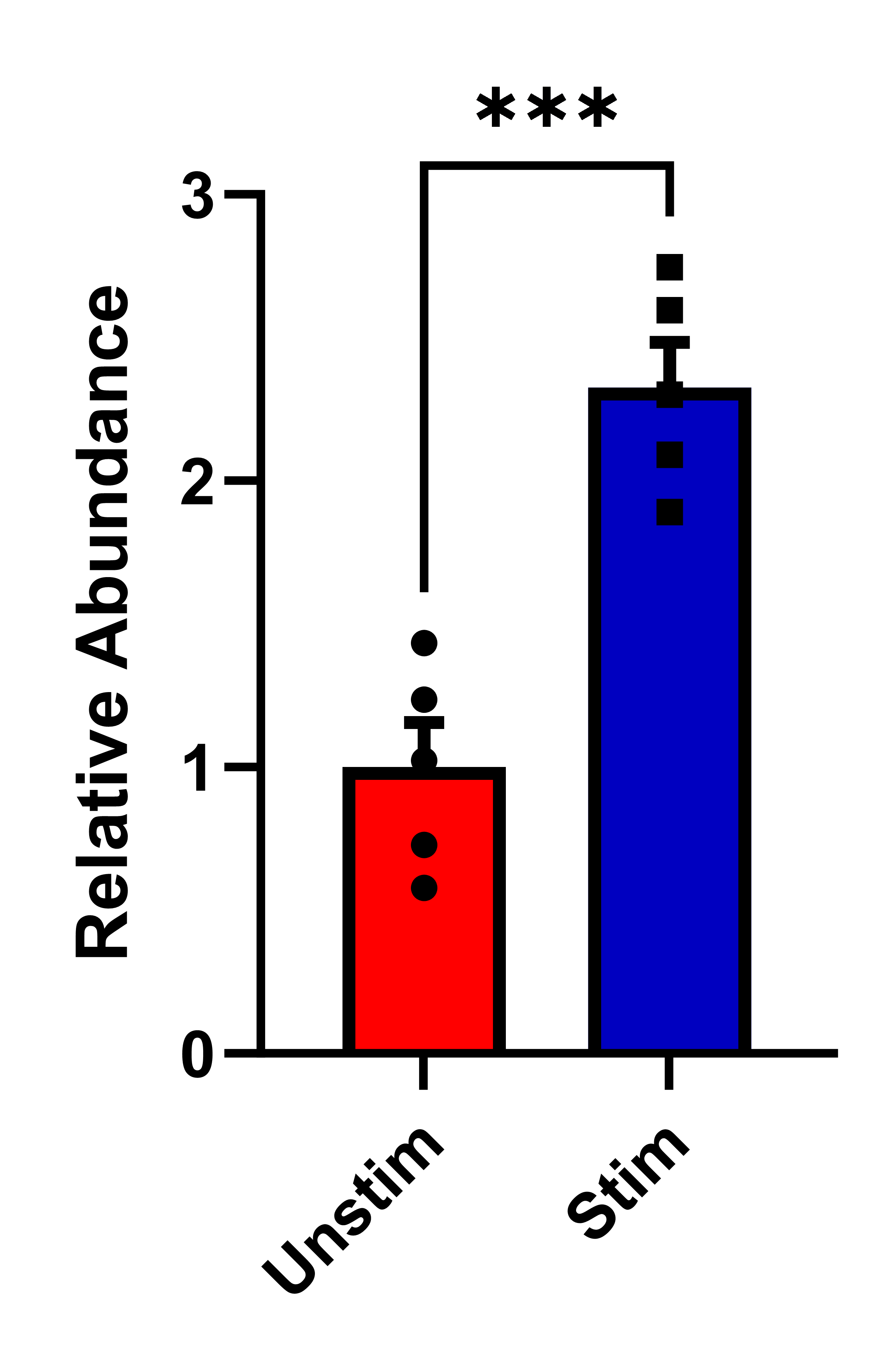

Supplement: Supplementary file 5 — Source Data Fig. 4 [file 44318_2024_30_MOESM5_ESM.zip › Figure 4/4B/Fig 4-B SXTBP-DDHD2 Interaction.tif]

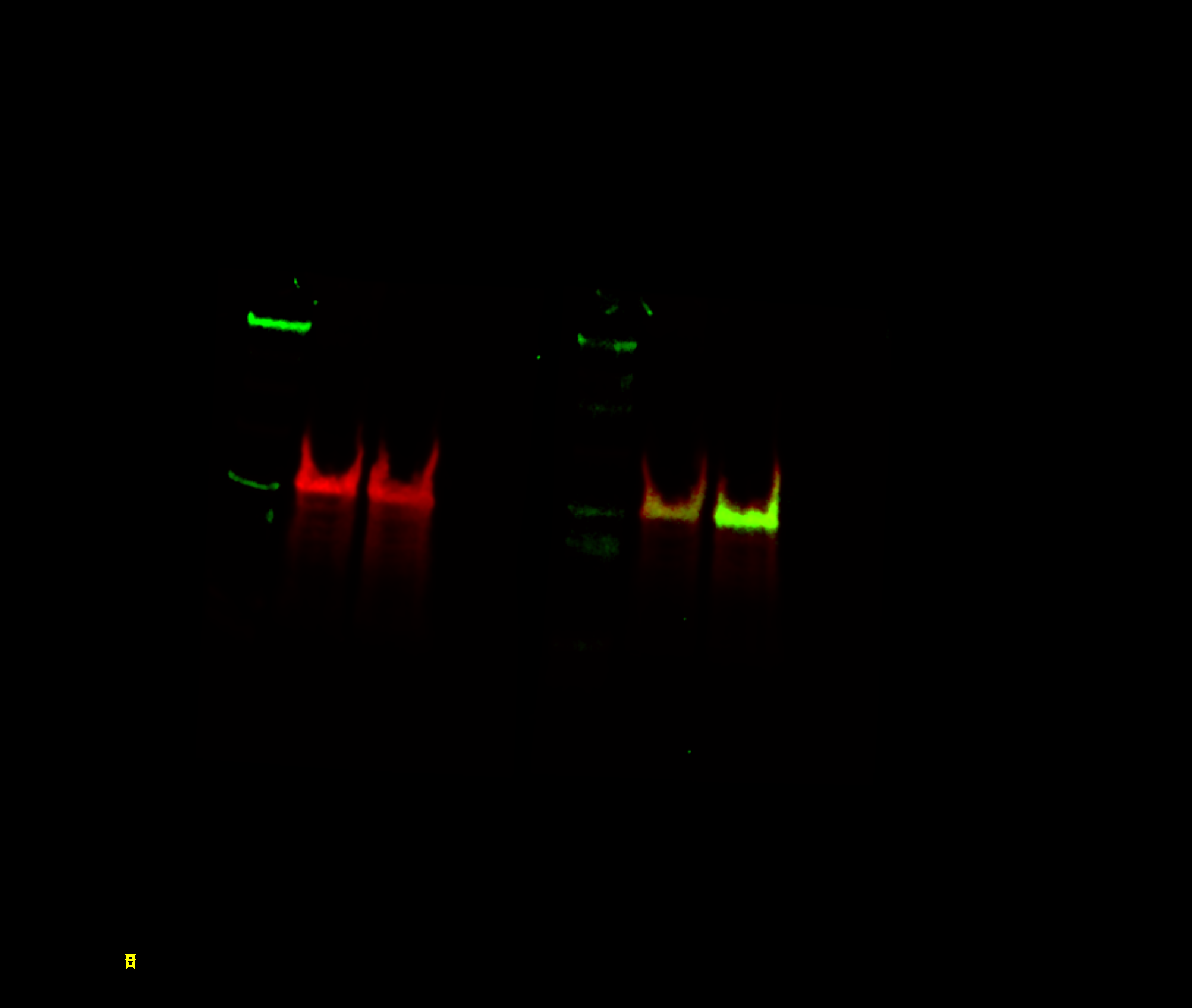

Supplement: Supplementary file 5 — Source Data Fig. 4 [file 44318_2024_30_MOESM5_ESM.zip › Figure 4/4C/Munc18-1-GFP-pulldown DDHD2.tif]

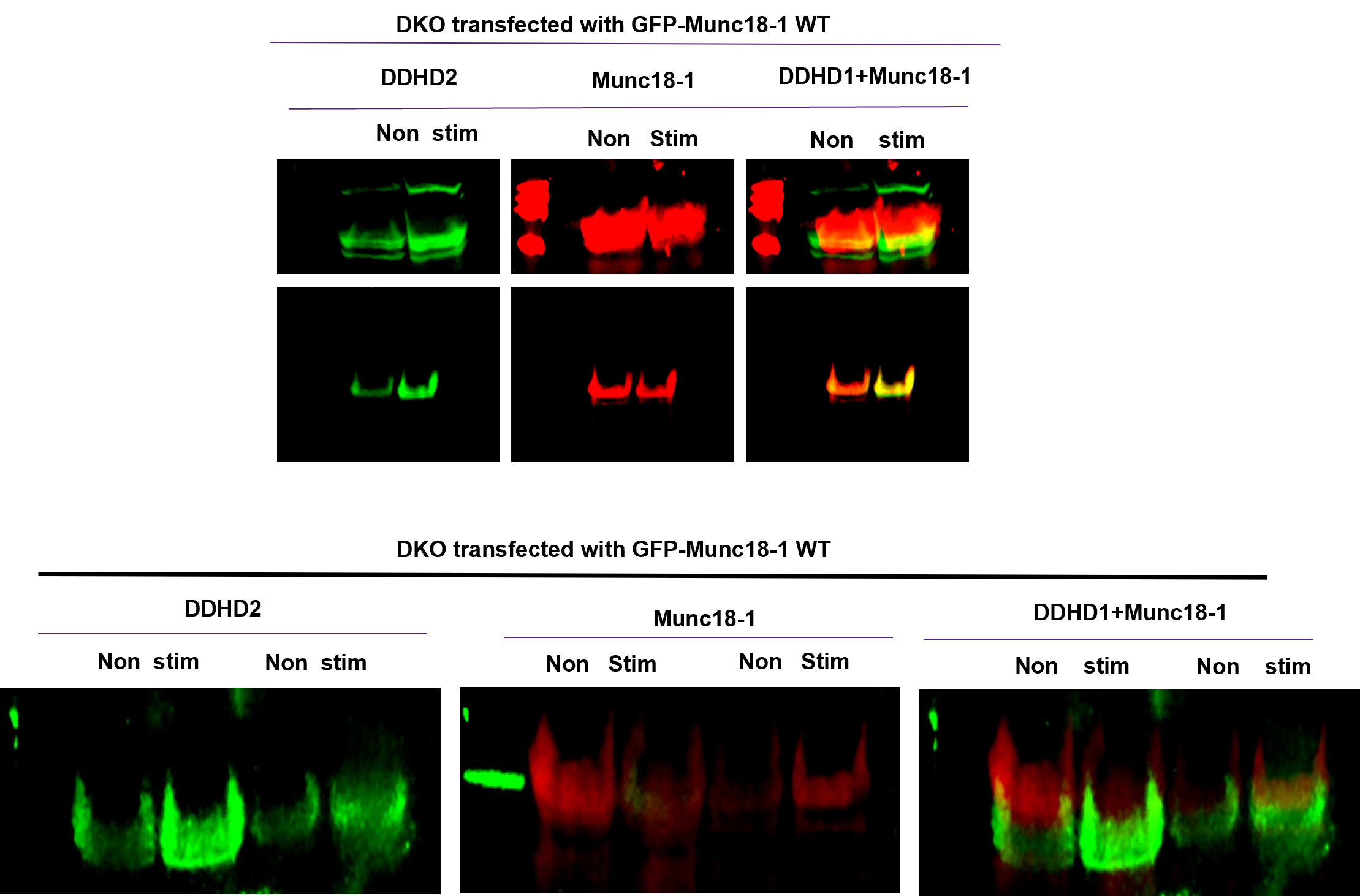

Supplement: Supplementary file 5 — Source Data Fig. 4 [file 44318_2024_30_MOESM5_ESM.zip › Figure 4/4C/Picture1.png]

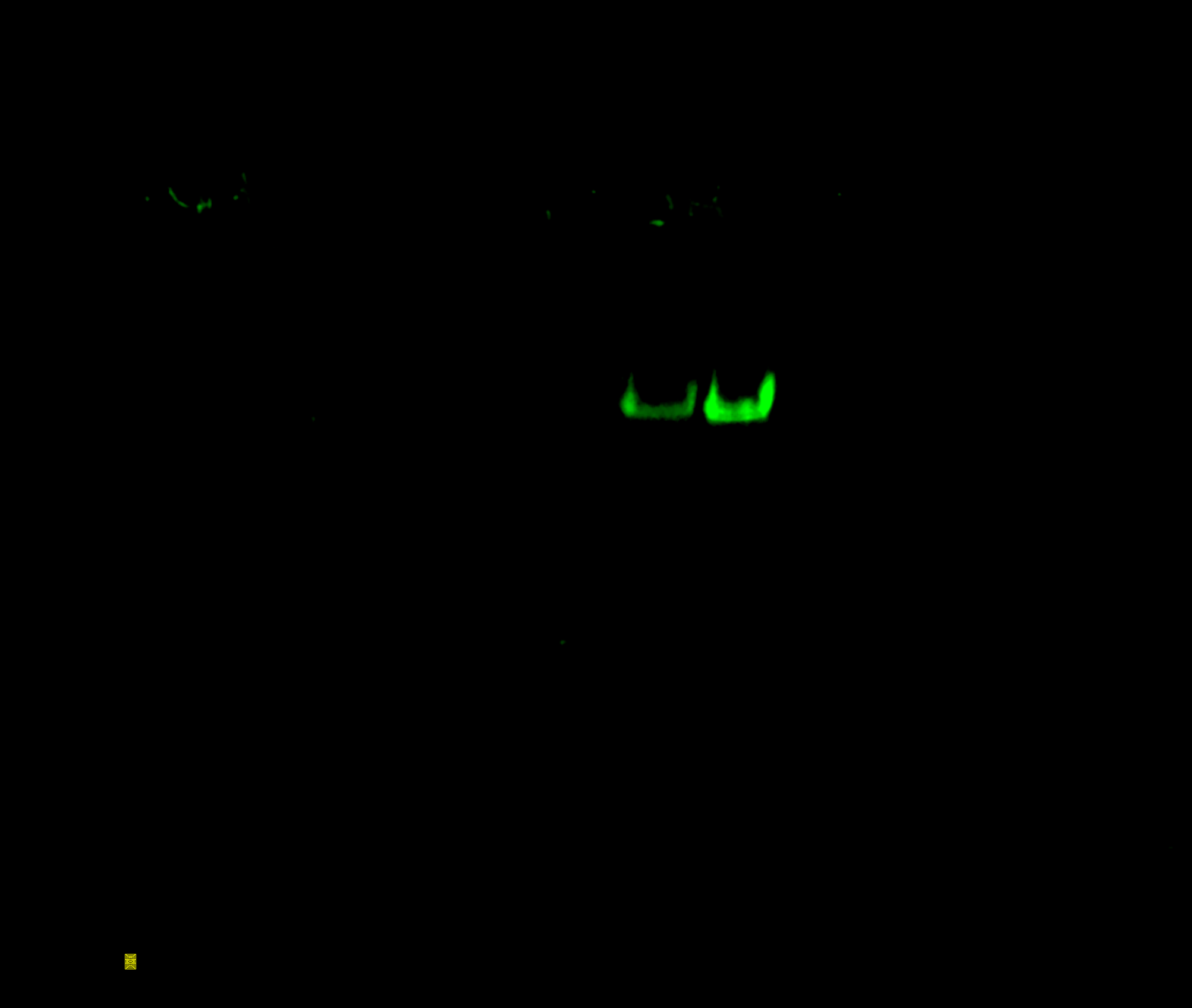

Supplement: Supplementary file 5 — Source Data Fig. 4 [file 44318_2024_30_MOESM5_ESM.zip › Figure 4/4C/Pull-down_repeated_2_DDHD2.tif]

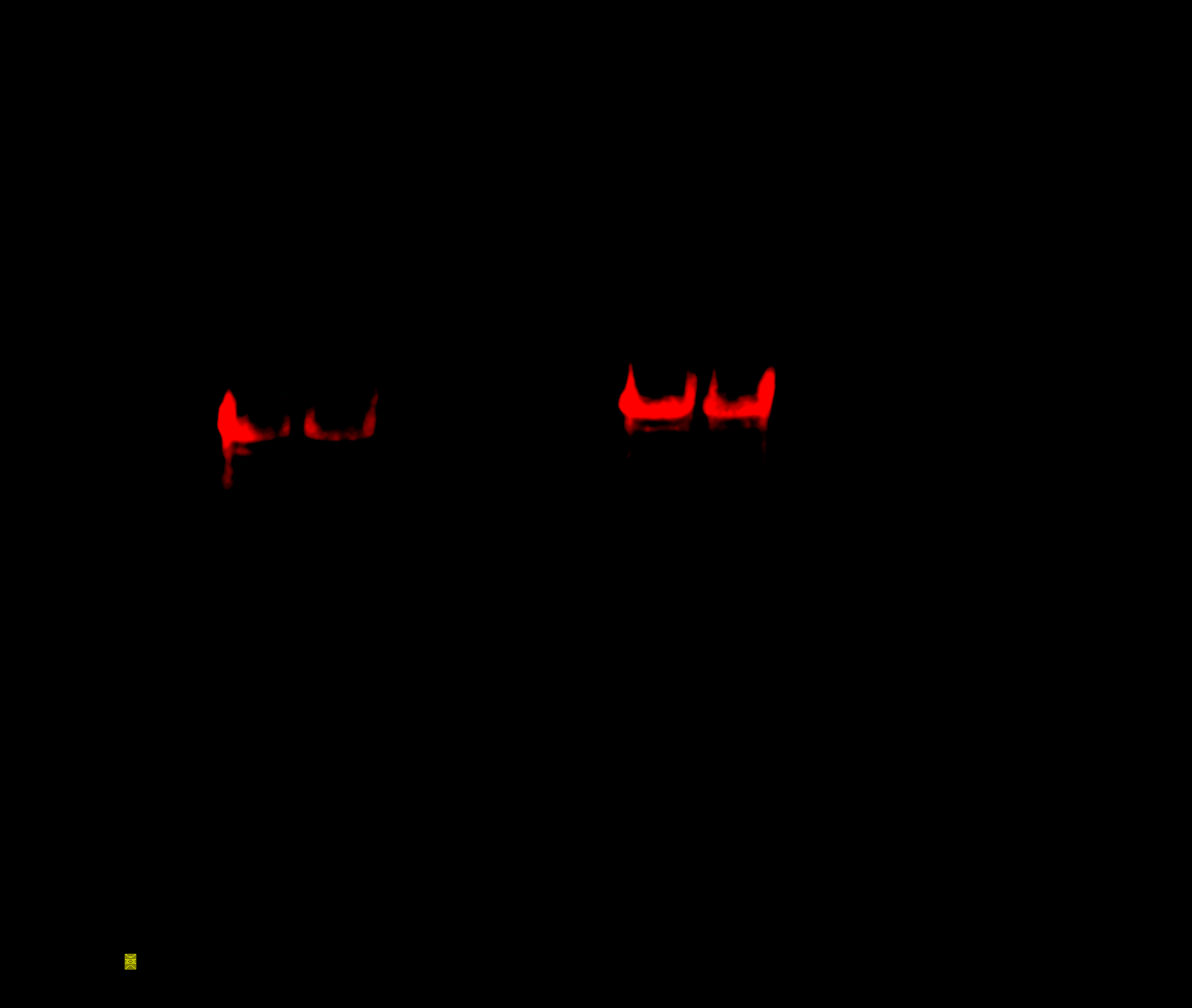

Supplement: Supplementary file 5 — Source Data Fig. 4 [file 44318_2024_30_MOESM5_ESM.zip › Figure 4/4C/Pull-down_repeated_2_Munc18_New.tif]

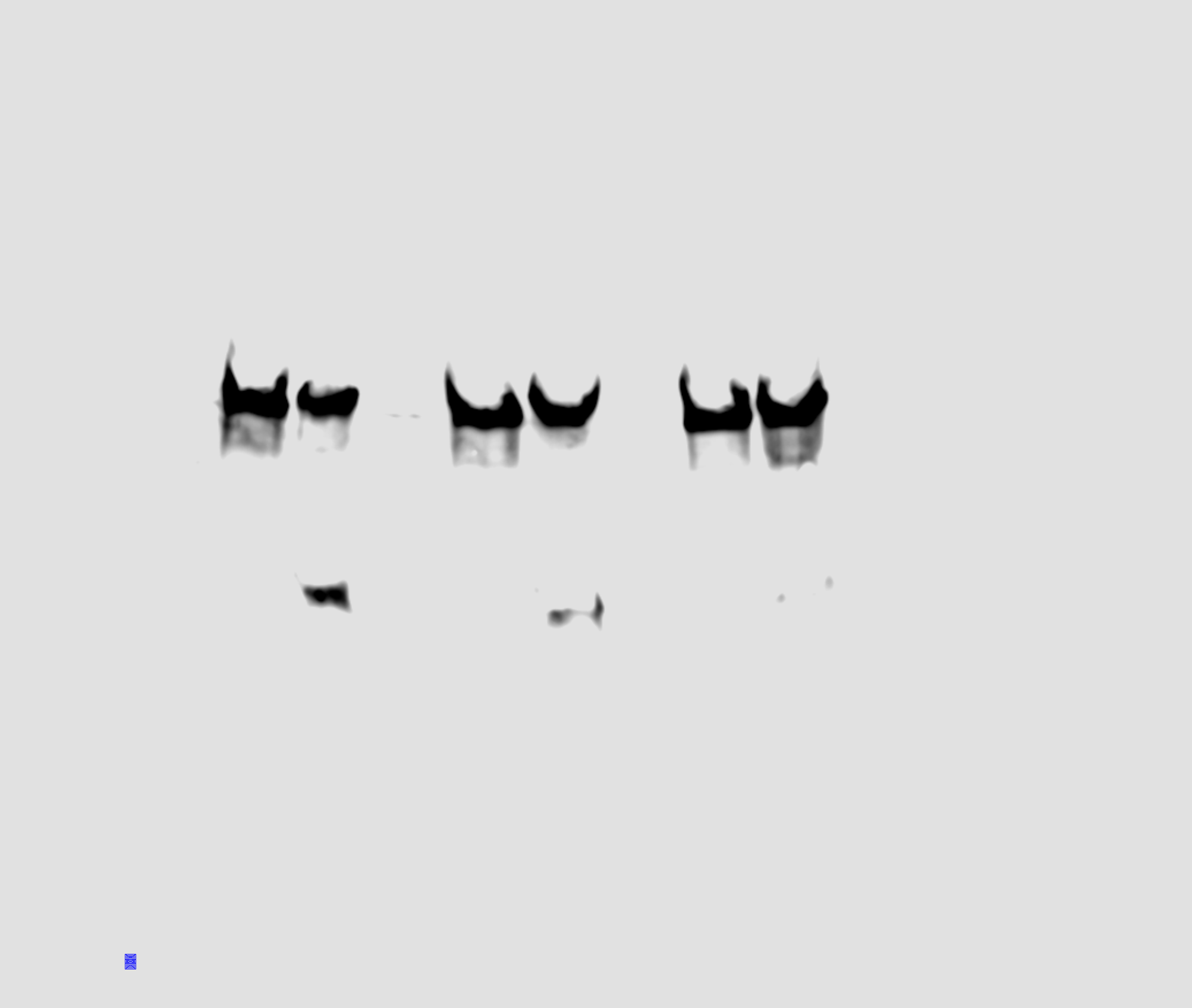

Supplement: Supplementary file 5 — Source Data Fig. 4 [file 44318_2024_30_MOESM5_ESM.zip › Figure 4/4D/DDHD2-GFP-Munc18-1.tif]

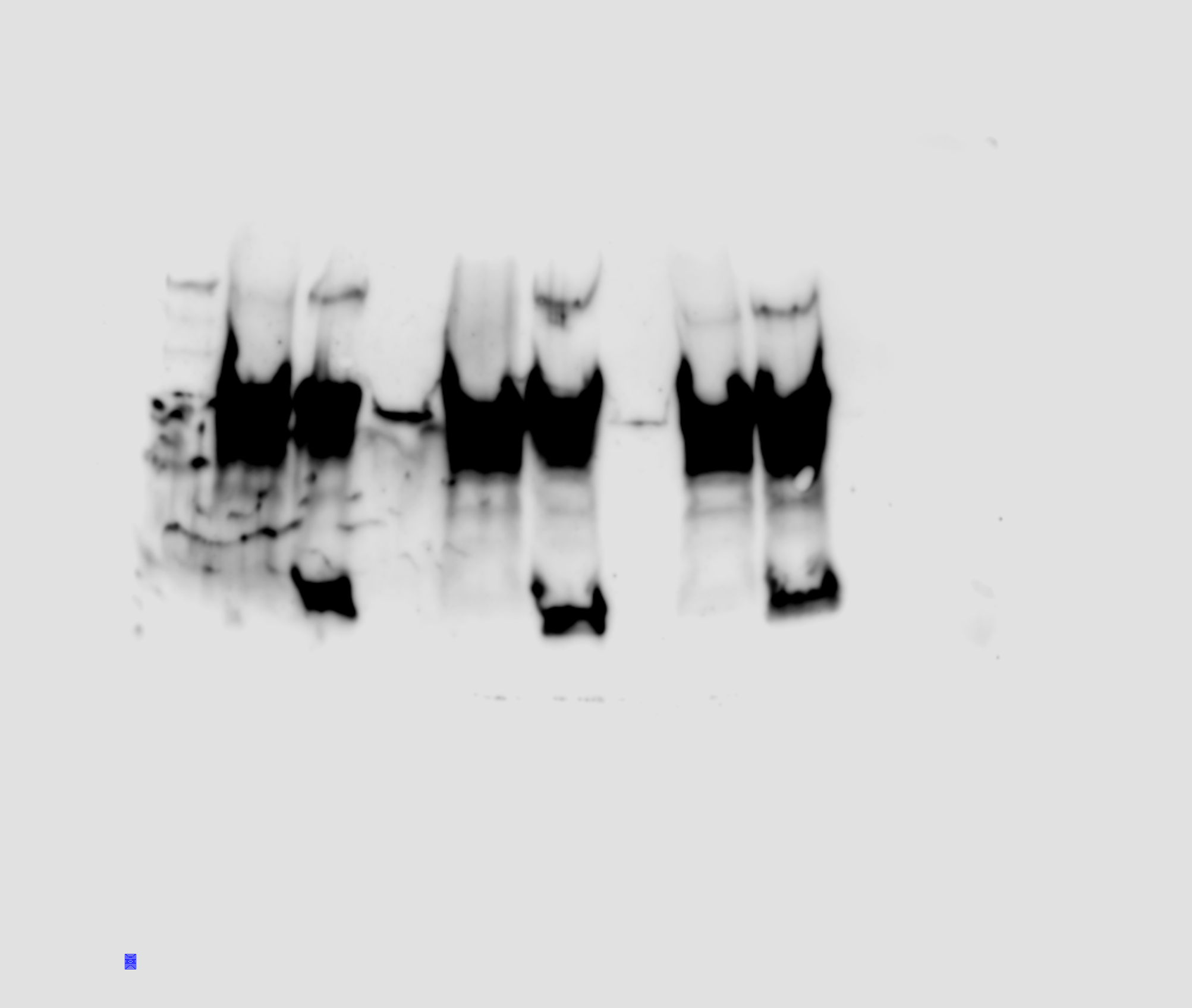

Supplement: Supplementary file 5 — Source Data Fig. 4 [file 44318_2024_30_MOESM5_ESM.zip › Figure 4/4D/DDHD2-GFP-pulldown-munc1805042022.tif]

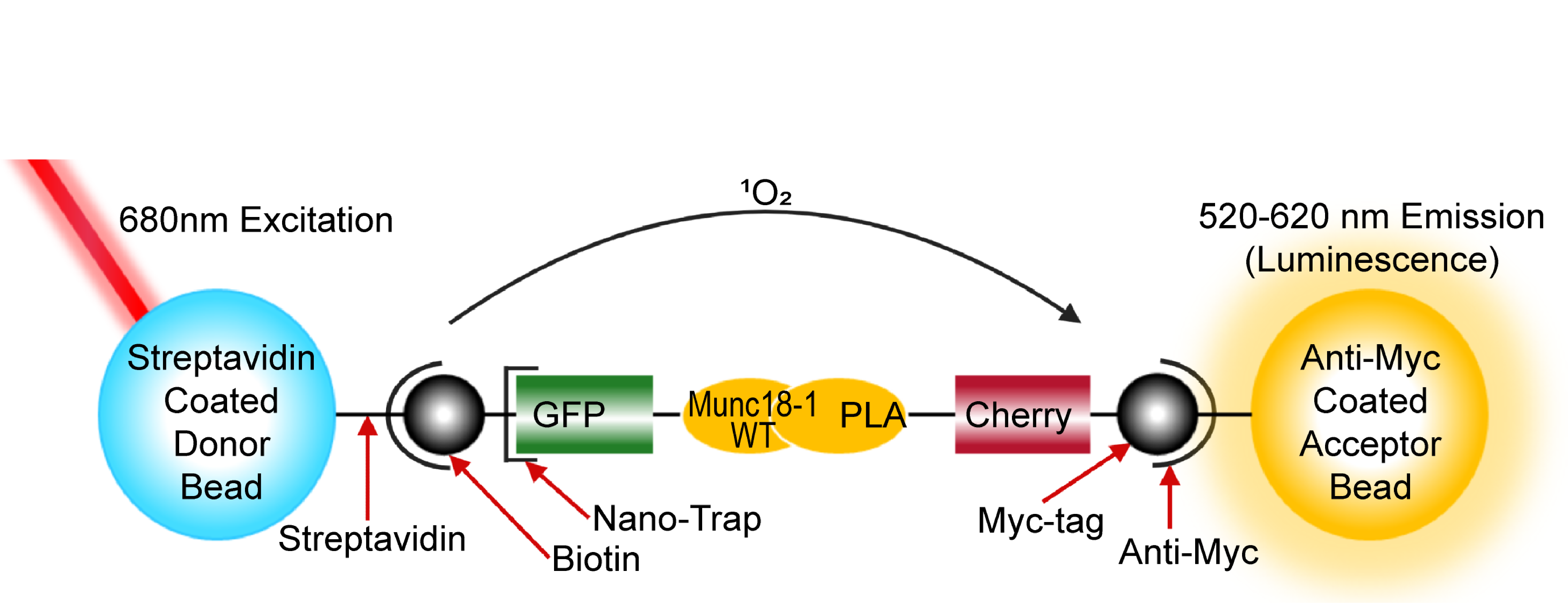

Supplement: Supplementary file 5 — Source Data Fig. 4 [file 44318_2024_30_MOESM5_ESM.zip › Figure 4/4E/Figure 4E Alphascreen illustration.tif]

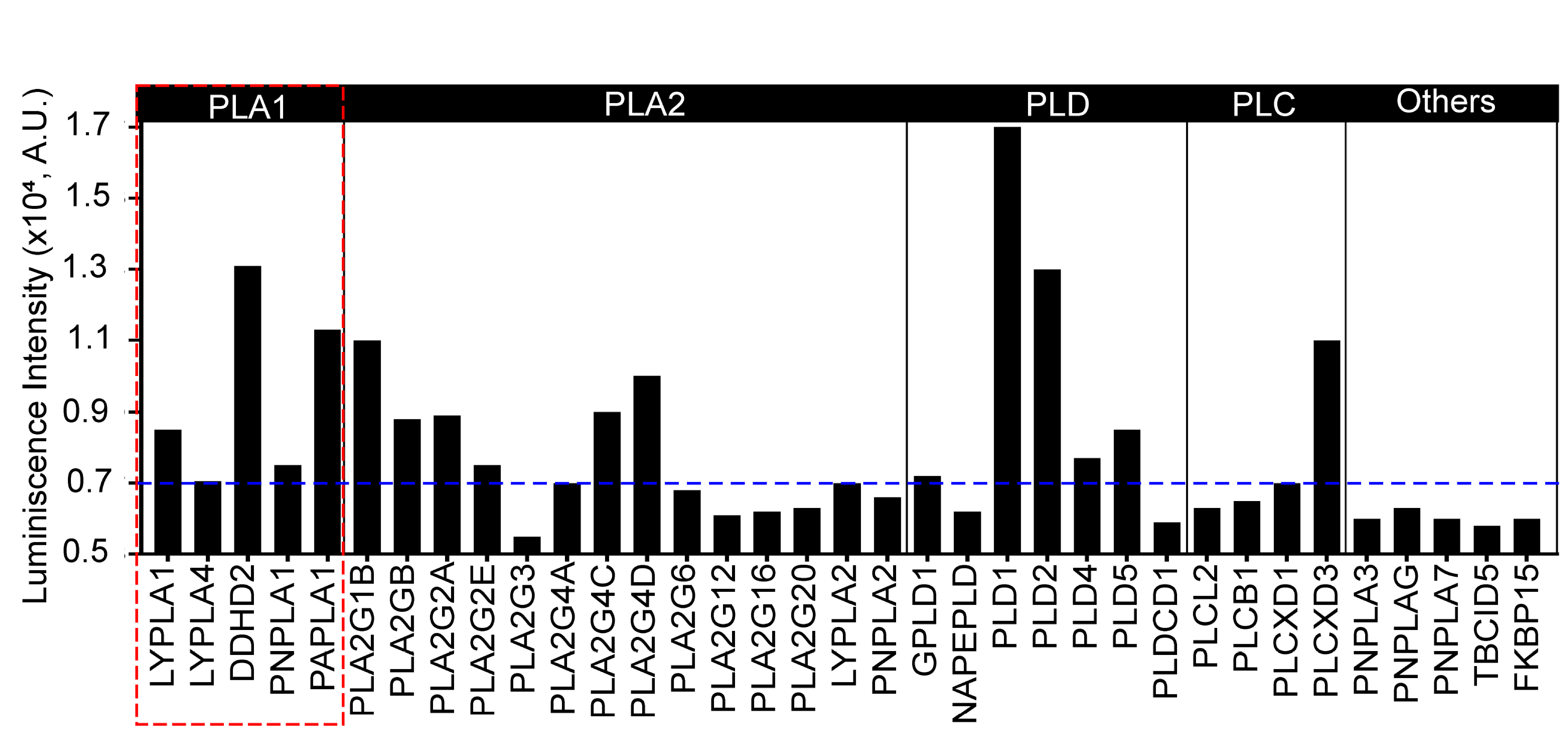

Supplement: Supplementary file 5 — Source Data Fig. 4 [file 44318_2024_30_MOESM5_ESM.zip › Figure 4/4F/Figure 4E Alphascreen.tif]

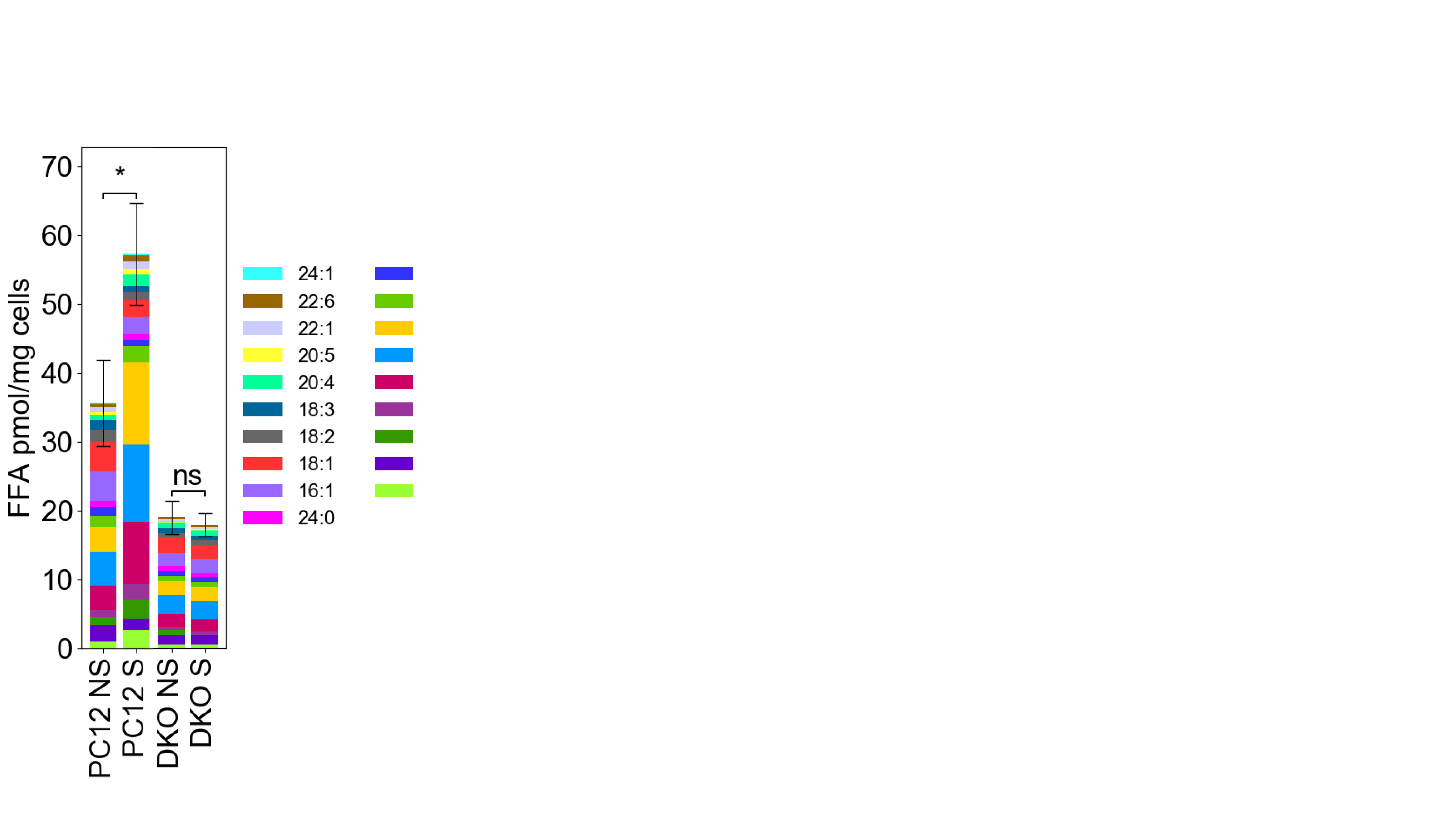

Supplement: Supplementary file 6 — Source Data Fig. 8 [file 44318_2024_30_MOESM6_ESM.zip › Figure 8/8A/Figure 8A FFA Profile in DKO PC12 Cells.tif]

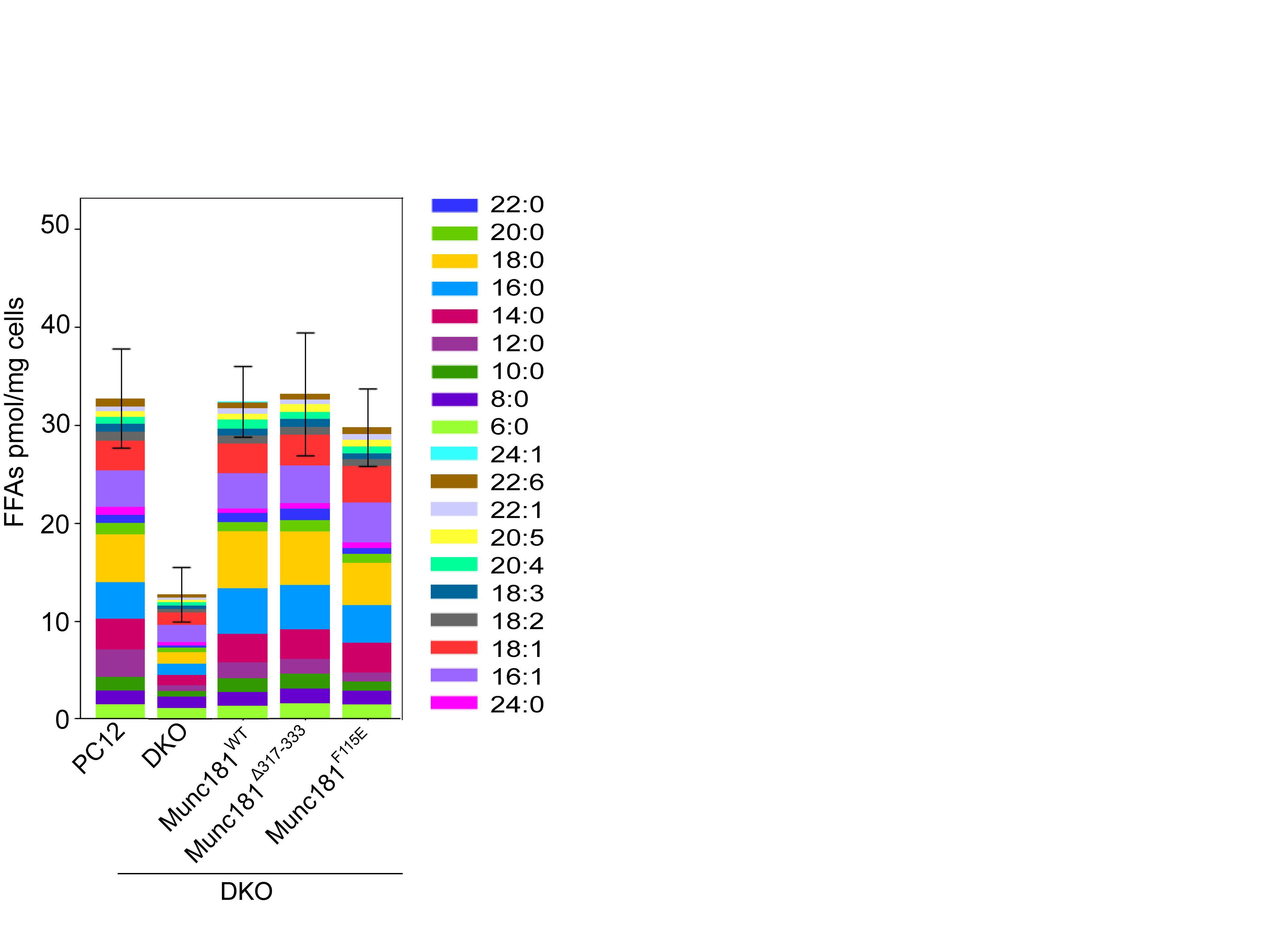

Supplement: Supplementary file 6 — Source Data Fig. 8 [file 44318_2024_30_MOESM6_ESM.zip › Figure 8/8B/Figure 8B FFA Profile in DKO Rescue.tif]

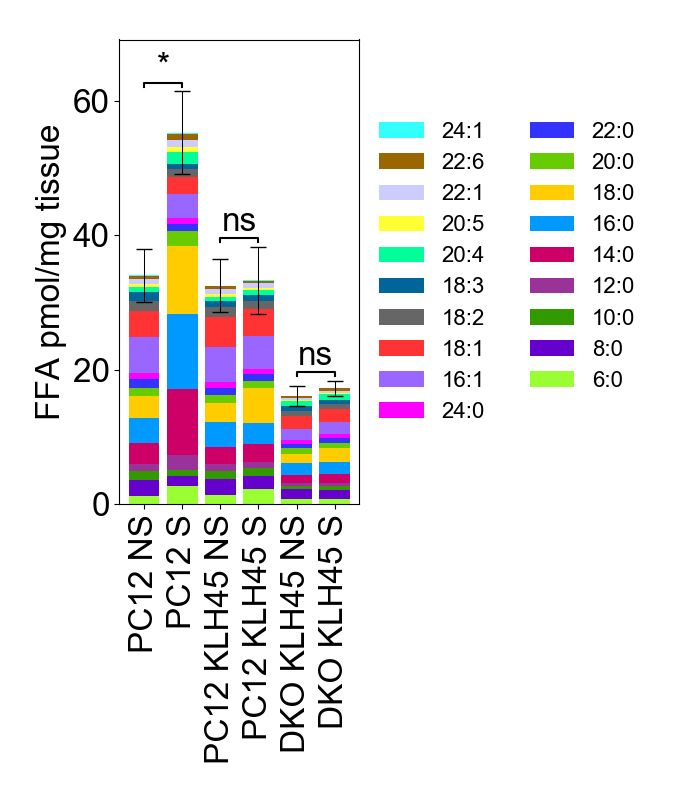

Supplement: Supplementary file 6 — Source Data Fig. 8 [file 44318_2024_30_MOESM6_ESM.zip › Figure 8/8C/Figure 8C KLH Inhibition of FFA.png]

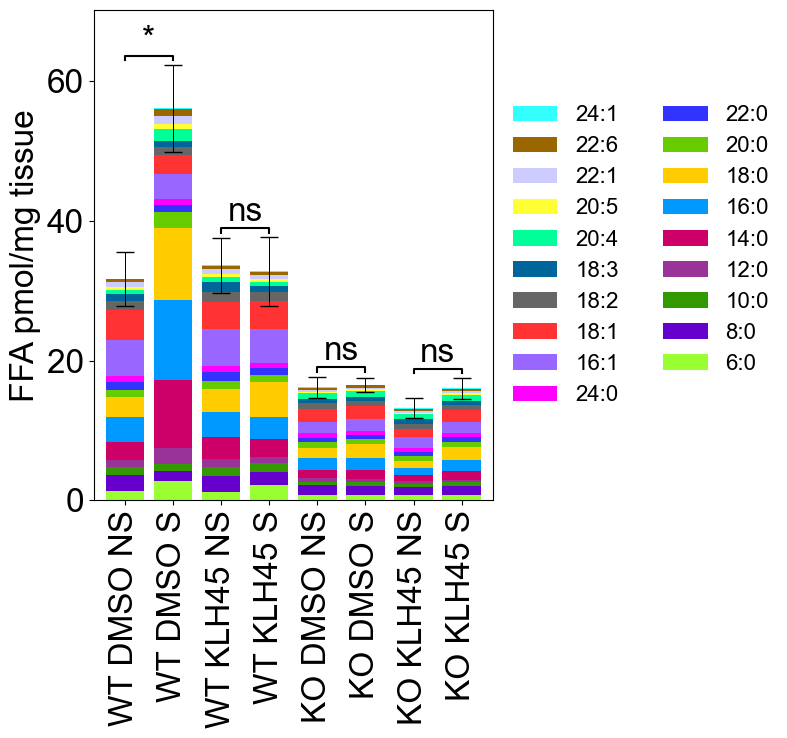

Supplement: Supplementary file 6 — Source Data Fig. 8 [file 44318_2024_30_MOESM6_ESM.zip › Figure 8/8D/Figure 8D Inhibition of FFA in Stimulated neuron.png]

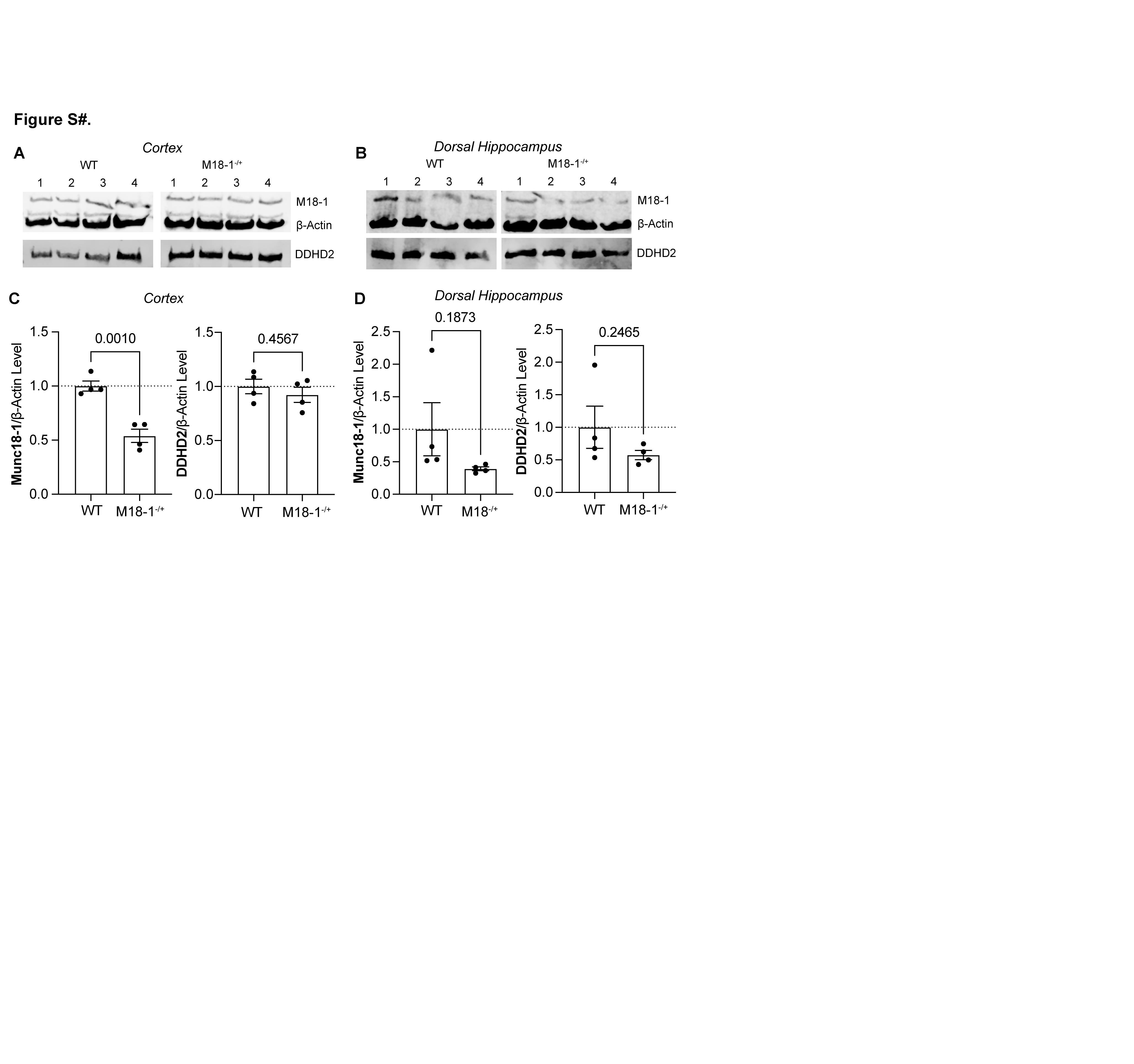

Supplement: Supplementary file 7 — Source Data Fig. 9 [file 44318_2024_30_MOESM7_ESM.zip › Figure 9/9A/Figure 9A Western blot of STXBP Haploinsufficient mice.tif]

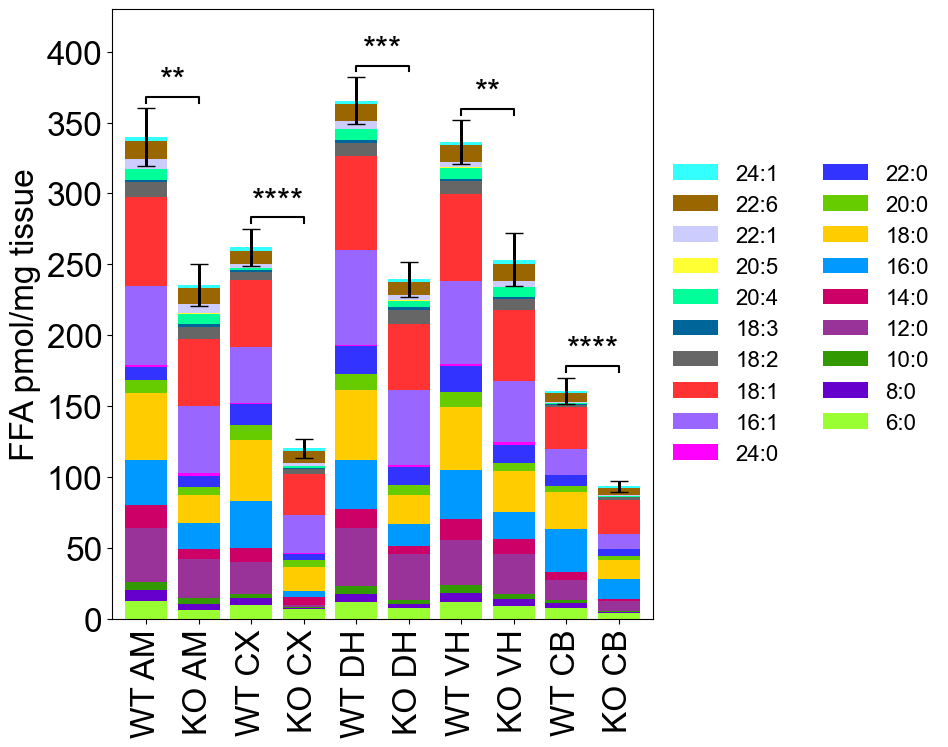

Supplement: Supplementary file 7 — Source Data Fig. 9 [file 44318_2024_30_MOESM7_ESM.zip › Figure 9/9C/20220701-145219_ffa_stackplot of STXBP Haploinsufficient mice.png]

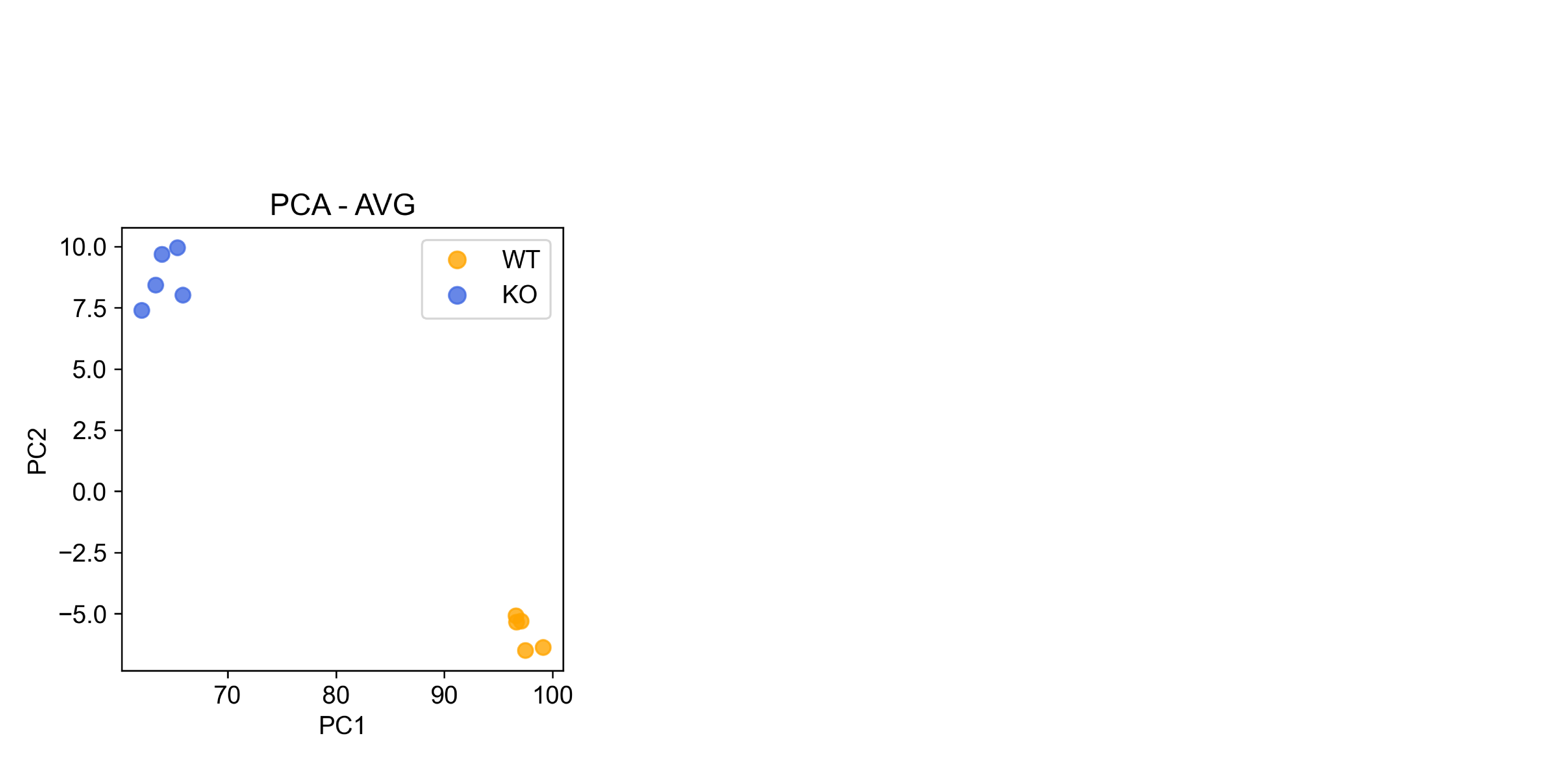

Supplement: Supplementary file 7 — Source Data Fig. 9 [file 44318_2024_30_MOESM7_ESM.zip › Figure 9/9D/Figure 9D PLSD FFA Changes.tif]

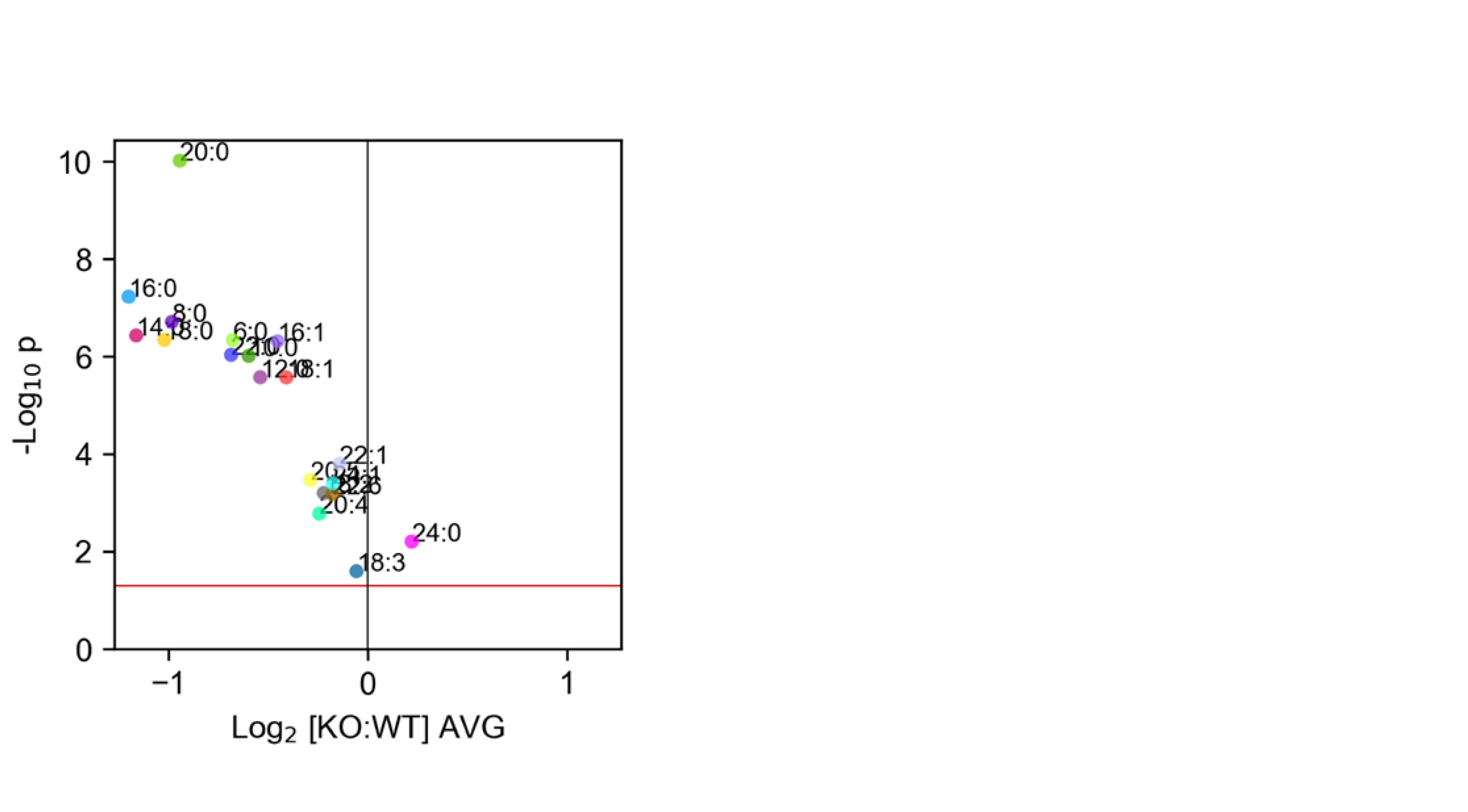

Supplement: Supplementary file 7 — Source Data Fig. 9 [file 44318_2024_30_MOESM7_ESM.zip › Figure 9/9E/Figure 9E Volcano plots of FFA in STXBP Haploinsufficient mice.tif]
