# Supplementary material for: The DDHD2-STXBP1 interaction mediates long-term memory via generation of saturated free fatty acids
Source: EMBO J. 2024 Feb 5;43(4):533–67. doi: 10.1038/s44318-024-00030-7 (PMC10897203; doi:10.1038/s44318-024-00030-7)
Supplement: Supplementary file 9 — Expanded View Figures [file 44318_2024_30_MOESM9_ESM.pdf]

Expanded View Figure

Figure EV1. Longitudinal assessment of motor function, spatial memory, and explorative behaviours in *DDHD2*$^{+/+}$ vs *DDHD2*$^{-/-}$ mice.

(**A**) Line graphs showing monthly recordings of a motor coordination, assessed using a rotarod device by determining the latency period from when the mouse is placed on the accelerating rotating rod device to the initial fall (s), and (**B**) motor strength (N), assessed using a grip strength device. Longitudinal monitoring of mouse activity with line graphs showing (**C**) vertical counts, (**D**) jump counts, (**E**) ambulatory distance (m), (**F**), vertical time (s), and (**G**) jump time (s). (**H**) Longitudinal assessment of spatial memory performance in mice, using the Novel Object Location (NOL) paradigm, represented as a line graph. Data information: In (**A–H**), the significance of the difference between each group as determined by one-way ANOVA ($n = 20$) is indicated by asterisks *$p < 0.05$, **$p < 0.01$, ***$p < 0.001$, ns = not significant. Error bars represent the cumulative standard error of the mean (SEM) for all groups and parameters.

 © The Author(s)

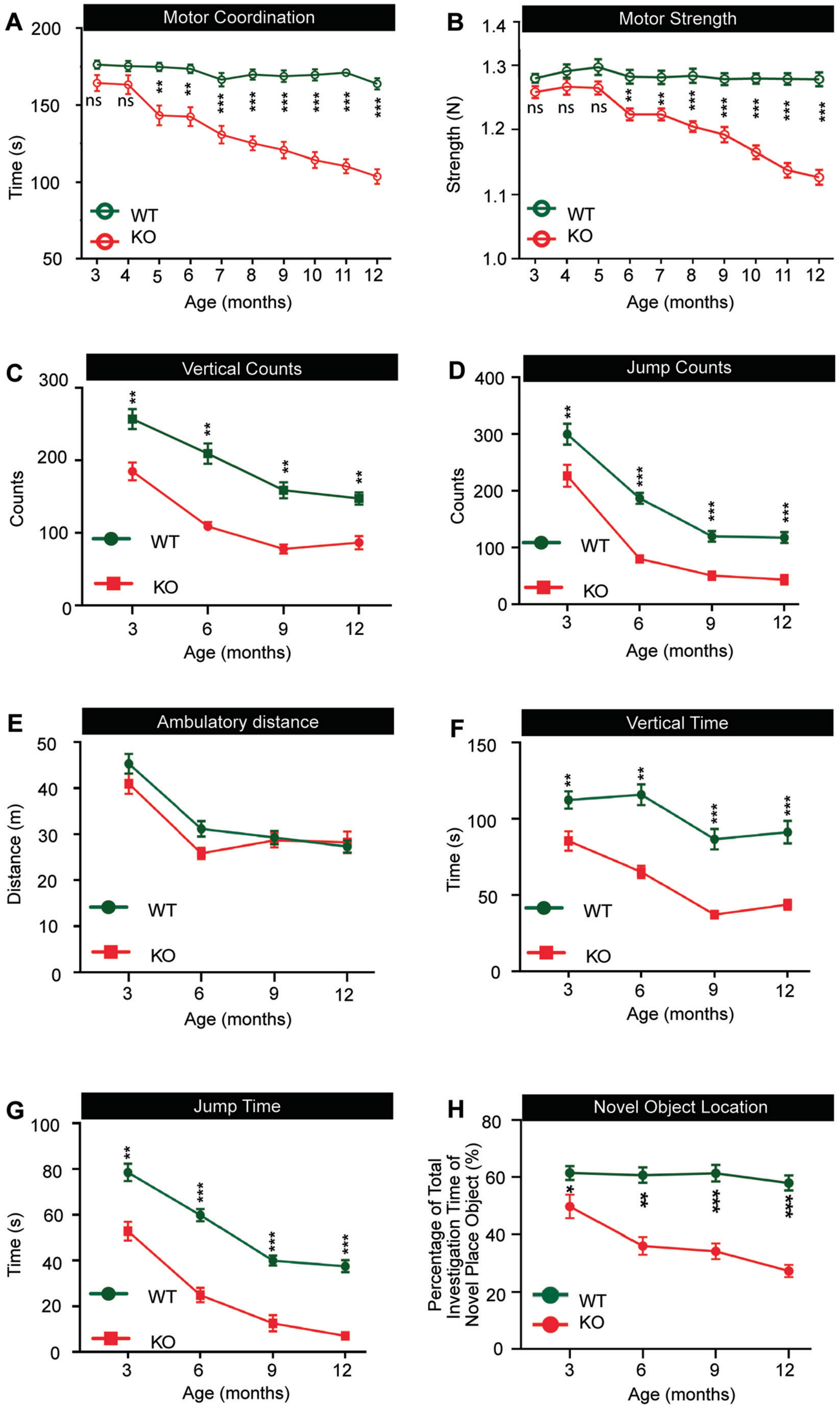
A
Motor Coordination
Time (s)
Age (months)
WT
KO
ns ns ** ** *** *** *** *** *** ***
B
Motor Strength
Strength (N)
Age (months)
WT
KO
ns ns ns ** ** *** *** *** *** ***
C
Vertical Counts
Counts
Age (months)
WT
KO
D
Jump Counts
Counts
Age (months)
WT
KO
E
Ambulatory distance
Distance (m)
Age (months)
WT
KO
F
Vertical Time
Time (s)
Age (months)
WT
KO
G
Jump Time
Time (s)
Age (months)
WT
KO
H
Novel Object Location
Percentage of Total Investigation Time of Novel Place Object (%)
Age (months)
WT
KO

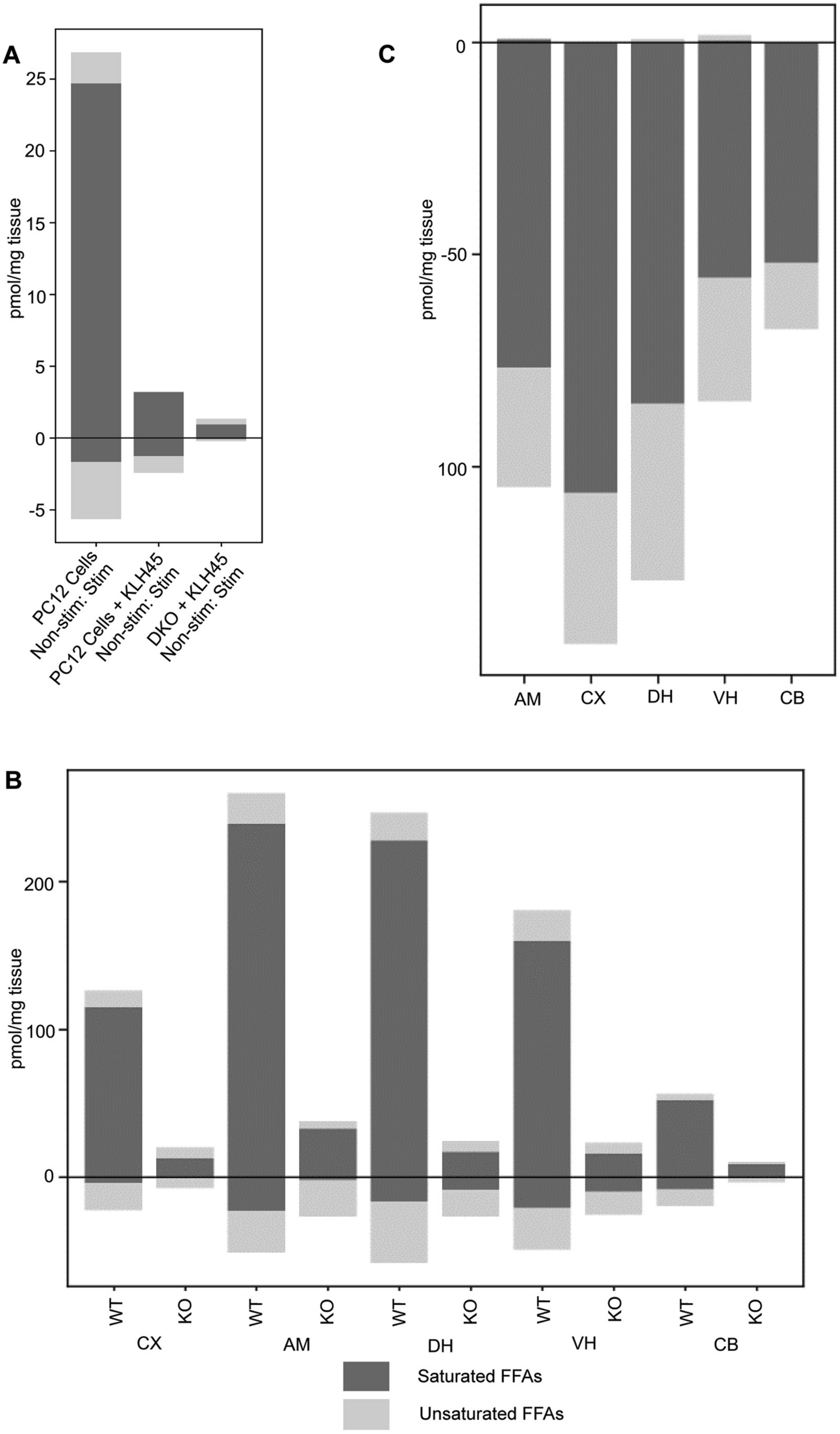
A
pmol/mg tissue
25
20
15
10
5
0
-5
PC12 Cells Non-stim: Stim
PC12 Cells + KLH45 Non-stim: Stim
DKO + KLH45 Non-stim: Stim
C
pmol/mg tissue
0
-50
100
AM
CX
DH
VH
CB
B
pmol/mg tissue
200
100
0
WT
KO
CX
AM
DH
VH
CB
Saturated FFAs
Unsaturated FFAs

 © The Author(s)

Figure EV2. Change in saturated (dark grey) vs unsaturated (light grey) FFAs in response to different conditions.

Bar plots show the change of saturated vs unsaturated FFAs in in response to (**A**) secretagogue stimulation in PC12 cells; (**B**) instrumental conditioning across the brain of WT and DDHD2 KO mice. (**C**) STXBP1 heterozygote across the brain versus WT.

© The Author(s)

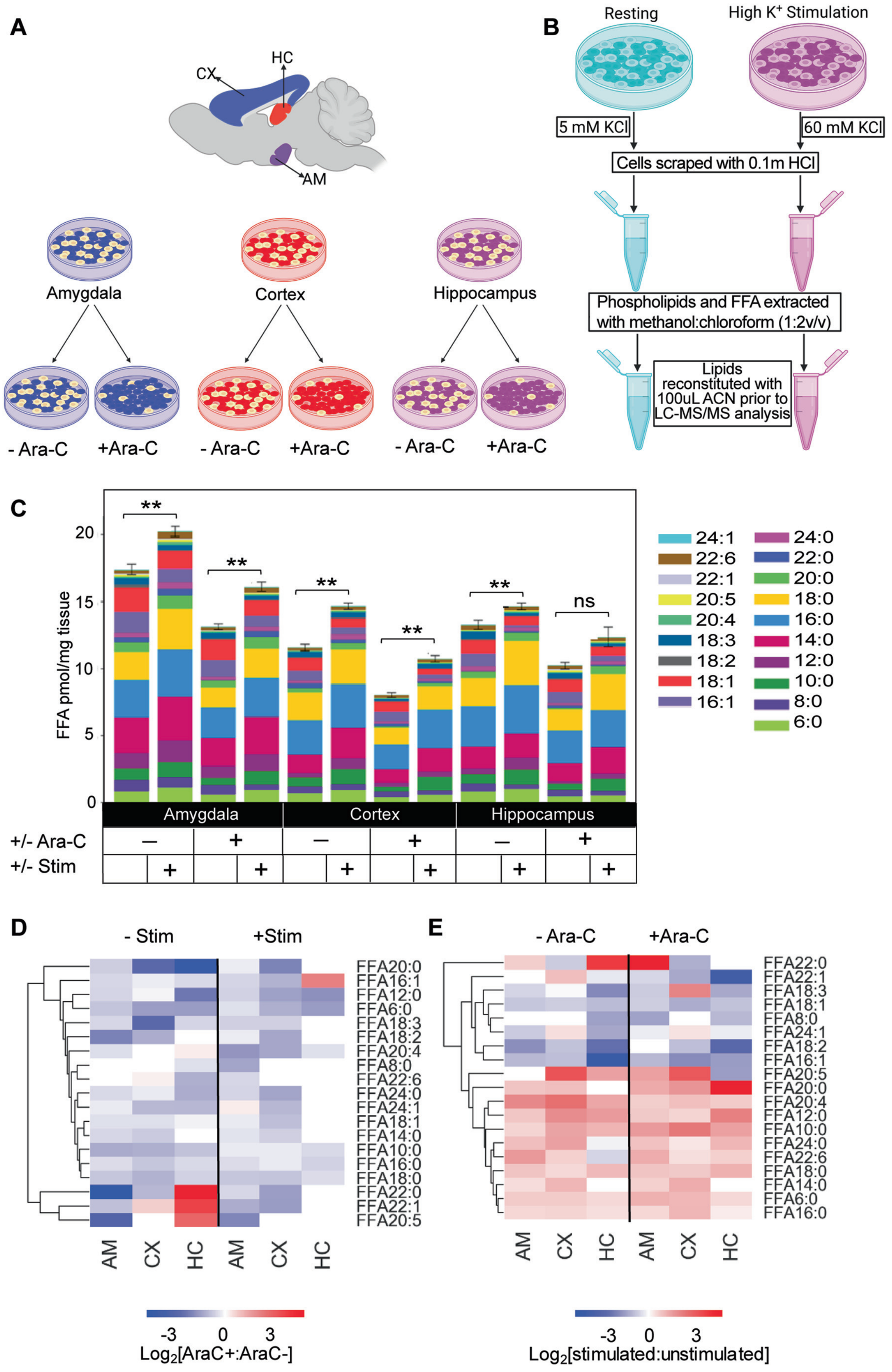
A
CX
HC
AM
Amygdala
Cortex
Hippocampus
- Ara-C
+Ara-C
- Ara-C
+Ara-C
- Ara-C
+Ara-C
B
Resting
High K+ Stimulation
5 mM KCl
60 mM KCl
Cells scraped with 0.1m HCl
Phospholipids and FFA extracted with methanol:chloroform (1:2v/v)
Lipids reconstituted with 100uL ACN prior to LC-MS/MS analysis
C
FFA pmol/mg tissue
**
**
**
**
**
ns
Amygdala
Cortex
Hippocampus
+/- Ara-C
+/- Stim
24:1
22:6
22:1
20:5
20:4
18:3
18:2
18:1
16:1
24:0
22:0
20:0
18:0
16:0
14:0
12:0
10:0
8:0
6:0
D
- Stim
+Stim
FFA20:0
FFA16:1
FFA12:0
FFA6:0
FFA18:3
FFA18:2
FFA20:4
FFA8:0
FFA22:6
FFA24:0
FFA24:1
FFA18:1
FFA14:0
FFA10:0
FFA16:0
FFA18:0
FFA22:0
FFA22:1
FFA20:5
AM
CX
HC
AM
CX
HC
-3 0 3
Log2[AraC+:AraC-]
E
- Ara-C
+Ara-C
FFA22:0
FFA22:1
FFA18:3
FFA18:1
FFA8:0
FFA24:1
FFA18:2
FFA16:1
FFA20:5
FFA20:0
FFA20:4
FFA12:0
FFA10:0
FFA24:0
FFA22:6
FFA18:0
FFA14:0
FFA6:0
FFA16:0
AM
CX
HC
AM
CX
HC
-3 0 3
Log2[stimulated:unstimulated]

 © The Author(s)

Figure EV3. Profile of the effect of ara-C on FFAs response to stimulated neuroexocytosis.

(**A**) Inhibition of glia proliferation in neuronal cultures using Ara-C. Cells from the cortex (CX), amygdala (AM), and hippocampus (HC) were isolated from embryonic-day-18 (E18) Sprague-Dawley rat embryos and seeded in separate wells from the indicated brain regions. (**B**) Potassium stimulation of neuroexocytosis and extraction of lipids. Cultured neurons were treated for 15 min using high potassium buffer; 60 mM K^+ (depolarised) or 2 mM K^+ (resting control). FFA and phospholipids were extracted in using methanol:chloroform using the liquid–liquid extraction protocol of Bligh and Dyer (Bligh and Dyer, 1959). (**C**) Bar graph showing quantification of FFA in Amygdala (AM), Cortex (CX), and Hippocampal (HC) neurons with and without ara-C treatment. (**D**) Hierarchical clustering heatmap showing FFA in response to ara-C treatment in stimulated versus non-stimulated cultures. (**E**) Hierarchical clustering heatmap showing FFA responses to stimulated neuroexocytosis with and without ara-C treatment.
Data information: In (**C**), the significance of the difference between each group ($n = 3$ biological replicates) as determined by unpaired *t*-test with Holm–Sidak post hoc correction is indicated by asterisks $^{**}p < 0.01$, ns = not significant. Error bars represent the cumulative standard error of the mean (SEM) for all groups and parameters.

© The Author(s)
